# Disarming the virulence arsenal of *Pseudomonas aeruginosa* by blocking two-component system signaling†
†Electronic supplementary information (ESI) available. See DOI: 10.1039/c8sc02496k


**DOI:** 10.1039/c8sc02496k

**Published:** 2018-07-10

**Authors:** Manibarsha Goswami, Adeline Espinasse, Erin E. Carlson

**Affiliations:** a Department of Chemistry , University of Minnesota , 225 Pleasant St. SE , Minneapolis , MN 55454 , USA . Email: carlsone@umn.edu; b Department of Medicinal Chemistry , University of Minnesota , USA; c Department of Biochemistry, Molecular Biology and Biophysics , University of Minnesota , USA

## Abstract

*Pseudomonas aeruginosa* infections have reached a “critical” threat status making novel therapeutic approaches required. Inhibiting key signaling enzymes known as the histidine kinases (HKs), which are heavily involved with its pathogenicity, has been postulated to be an effective new strategy for treatment. Herein, we demonstrate the potential of this approach with benzothiazole-based HK inhibitors that perturb multiple virulence pathways in the burn wound *P. aeruginosa* isolate, PA14. Specifically, our compounds significantly reduce the level of toxic metabolites generated by this organism that are involved in quorum-sensing and redox-balancing mechanisms. They also decrease the ability of this organism to swarm and attach to surfaces, likely by influencing their motility appendages. Quantitative transcription analysis of inhibitor-treated cultures showed substantial perturbations to multiple pathways including expression of response regulator GacA, the cognate partner of the “super regulator” of virulence, HK GacS, as well as flagella and pili formation. These promising results establish that blocking of bacterial signaling in *P. aeruginosa* has dramatic consequences on virulence behaviours, especially in the context of surface-associated infections.

## Abstract 2

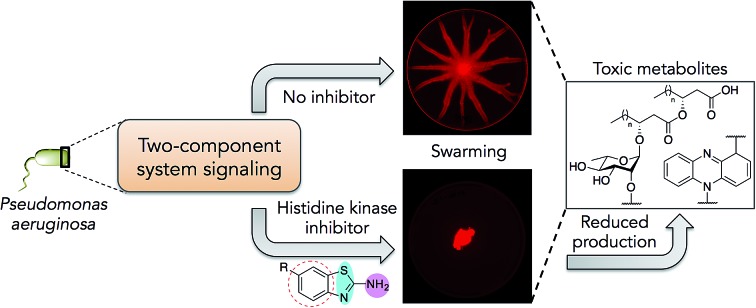
 *Pseudomonas aeruginosa* infections have reached a “critical” threat status making novel therapeutic approaches required.

## Introduction

**Fig. 1:**
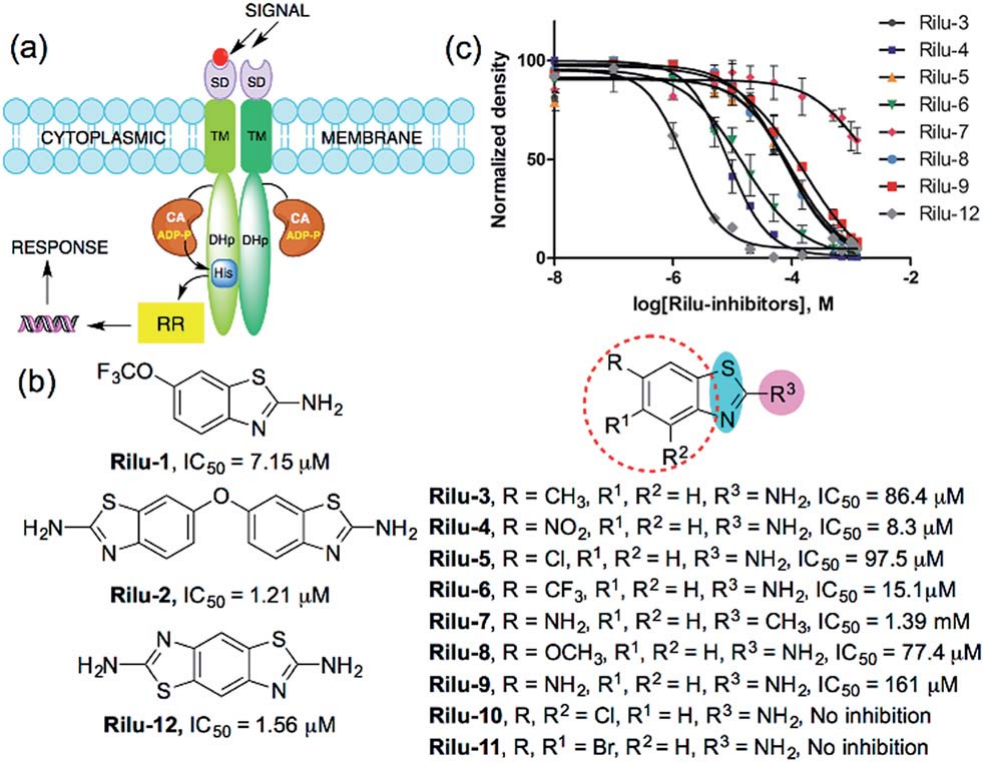
(a) TCS signaling pathway; transmembrane protein, HK and phosphoryl transfer to its cognate RR. Domains present in transmembrane HK portion; SD = sensor, TM = transmembrane, CA = catalytic and ATP-binding, DHp = dimerization and histidine phosphotransfer. His = histidine, ADP-P = adenosine triphosphate, (b) structures of compounds and their IC_50_ values for HK853 inhibitory activity, (c) dose–response curves of the compounds for HK inhibition (Fig. S1, Table S1†).

Bacterial virulence mechanisms have become promising new targets for the development of antibacterial agents, including ubiquitous signaling pathways that play key roles in many of these processes, the two-component systems (TCSs).^1^–^4^ Most bacteria rely heavily on TCSs; (>20 distinct TCSs per organism) to respond to their external environment and transmit various cell signals.^5^ TCSs are typically composed of a membrane-bound sensory protein, a histidine kinase (HK), and a cognate response regulator (RR) protein (Fig. 1). HKs respond to external stimuli by autophosphorylation, using ATP, of a catalytic histidine residue in the DHp (dimerization and histidine phosphotransfer) region and subsequent phosphoryl group transfer to the RR, which is often a transcription factor and induces altered gene expression (Fig. 1).^6^ TCSs and their corresponding HKs are postulated to be critical in many bacterial survival, metabolic, and virulence mechanisms.^1^–^4^,^7^ For example, HKs have been linked to severe infections caused by Gram-positive and Gram-negative bacteria, including the ESKAPE pathogens (*Enterococcus faecium*, *Staphylococcus aureus*, *Klebsiella pneumoniae*, *Acinetobacter baumannii*, *Pseudomonas aeruginosa*, and *Enterobacter* species).^8^ Antibiotic resistance has also been attributed to TCS signaling – for example, resistance to cationic antimicrobial peptides is thought to be associated with PhoQ/PhoP in *Salmonella* and GraS/GraR in *S. aureus*, whereas CzcR/CzcS is implicated in carbapenem-resistance in *P. aeruginosa.*^7^ While some TCSs are essential, most are non-essential and are quintessential in virulence processes in Gram-negative bacteria such as quorum sensing (QS), toxin production, and formation of bacterial communities.^2^,^7^ In light of these advantages, HKs have attained considerable interest as antivirulence targets.^2^,^7^

## Results and discussion

We postulated that targeting the highly-conserved catalytic ATP-binding (CA) domain within these proteins would be broadly applicable to a range of bacteria, and that simultaneous inhibition of multiple HKs within an organism would decrease its ability to quickly evolve resistance.^7^,^9^ In addition, HKs utilize a Bergerat fold to bind this substrate, which is not found in eukaryotic kinases, potentially providing a means for the generation of selective inhibitors.^7^,^9^ Recently, we reported nine lead compounds, identified from a high-throughput screen (HTS), which bind to the CA domain of multiple HKs.^9^ Two leads from the HTS share a common scaffold, a benzothiazole core, making this structure attractive for further study (Fig. 1b; **Rilu-1** and **2**).^9^ One of these compounds, riluzole (**Rilu-1**; IC_50_ = 7.2 μM) is an FDA approved amyotrophic lateral sclerosis drug, indicating that this chemotype has drug-like properties.^10^ In this work, we sought to assess the ability of these potent inhibitors to affect bacterial signaling in live cells and lay the foundation for the development of potent antivirulence agents.

**Rilu-1** has a trifluoromethoxy (OCF_3_) group at the 6-position on the ring and based on this core scaffold, we generated a small library of compounds to evaluate their biochemical potency and to provide a diversity of molecules for in cell assessment (**Rilu-3–11**, Fig. 1b and c). Compounds containing an electron-withdrawing group, such as –OCF_3_, –NO_2_, –CF_3_ (**Rilu-1**, **4**, **6**; 7–15 μM), were found to be potent inhibitors of HK autophosphorylation (HK853, Fig. 1b), while a mildly deactivating –Cl group, decreased potency (**Rilu-5**; 98 μM). Electron-donating groups such as –OCH_3_, –NH_2_ or even a weakly donating moiety such as –CH_3_ yielded ∼10-fold increase in the IC_50_ values (**Rilu-3**, **8**, **9**; 77–161 μM). Interestingly, functionalization with multiple deactivating groups yielded inactive compounds (**Rilu-10**, **11**). Recently, we demonstrated that a –N–NH–N– or similar triad, such as the –S–NH_2_–N–, is critical for CA domain binding (highlighted in cyan and pink, Fig. 1b).^11^ Consistent with this, we found that the positional isomer in which the –NH_2_ and –CH_3_ groups are switched shows minimal activity (comparison of **Rilu-3** and **7**, IC_50_ = 1.4 mM). The more potent lead from the HTS, **Rilu-2**, contains two benzothiazole rings, likely promoting additional polar interactions within the active site. Assessment of **Rilu-12**, which still features two amino moieties but does so in a rigid tricyclic structure, revealed it to be equivalently potent (IC_50_ 1.6 μM; Fig. 1b and c). From our HTS pilot screen library, ∼10 compounds containing a similar scaffold were “hits”, but several were ultimately found to be either low-binders or protein-aggregators (Fig. S2†).^9^ Thus, we selected four compounds (**Rilu-1**, **2**, **4**, **12**) containing one or two thiazole rings that were the most potent in our biochemical assay, as well as non-aggregators (Fig. S3†),^9^ and investigated their effects in live cells.

**Fig. 2:**
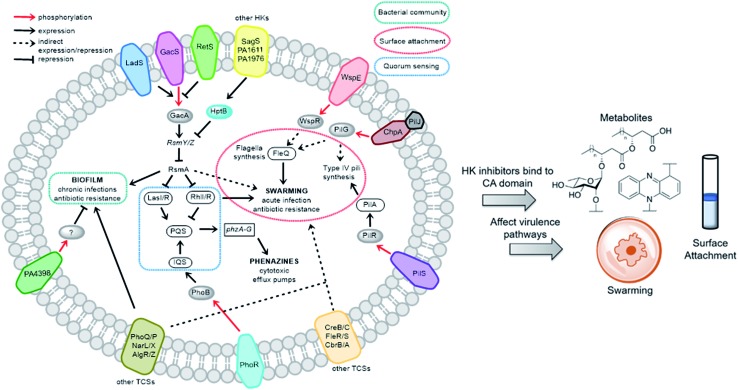
Important virulence pathways in *P. aeruginosa* regulated by various TCSs. Blocking these signaling networks with HK inhibitors can affect virulence mechanisms. The transmembrane proteins (different colours) indicate assorted HKs and their cognate RRs are shown as grey ovals. The legends of the arrows and boxes are indicated within the figure. Note: not all *P. aeruginosa* strains possess all of the HKs listed above. For example, PA14 does not have LadS, whereas this protein is found in the commonly studied lab strain, PAO1.

We sought to study the opportunistic Gram-negative organism *P. aeruginosa*, one of the most prevalent nosocomial pathogens and named on the most “critical” threats list by the World Health Organization.^12^ It is the leading cause of death associated with immune-compromised and CF patients. The virulent *P. aeruginosa* strain PA14, obtained from a burn wound, encodes a large number of TCS regulatory proteins in its genome, with >64 sensor HKs and >72 RRs.^13^ Among these HKs, many are intricately linked and influence virulence and antibiotic-resistance mechanisms such as biofilm formation, swarming, and toxin production (Fig. 2). These traits strongly suggest that inhibitors of these TCSs or HKs could provide viable leads for treatment of *P. aeruginosa* infection. Given our stated goal of identifying antivirulence agents, we first assessed **Rilu-1**, **2**, **4**, **12** for bacteriostatic and bactericidal properties in PA14 cultures. None of these compounds substantially slowed growth or killed the organism as determined both by OD_620_ measurements (Fig. S4†) and CFU studies (data not shown), even at high concentrations (500 μM). Given that some thiazoles have been reported to have off-target effects,^14^ we tested our inhibitors against a mammalian heat-shock protein (HSP90α), which possesses a similar ATP-binding Bergerat fold. None of the compounds were found to substantially inhibit this protein, even at concentrations of 1.25 mM (Fig. S5†). We have also demonstrated that **Rilu-1**, **2** are not cytotoxic to Vero cells at <180 μM in earlier studies.^9^ Recently, molecules containing a similar scaffold, 2-aminoimidazole, were reported as inhibitors of RR QseB that led to reduced biofilms in *Francisella*,^15^ further exemplifying the potential of this general class of compounds as promising antivirulence candidates.^16^

**Fig. 3:**
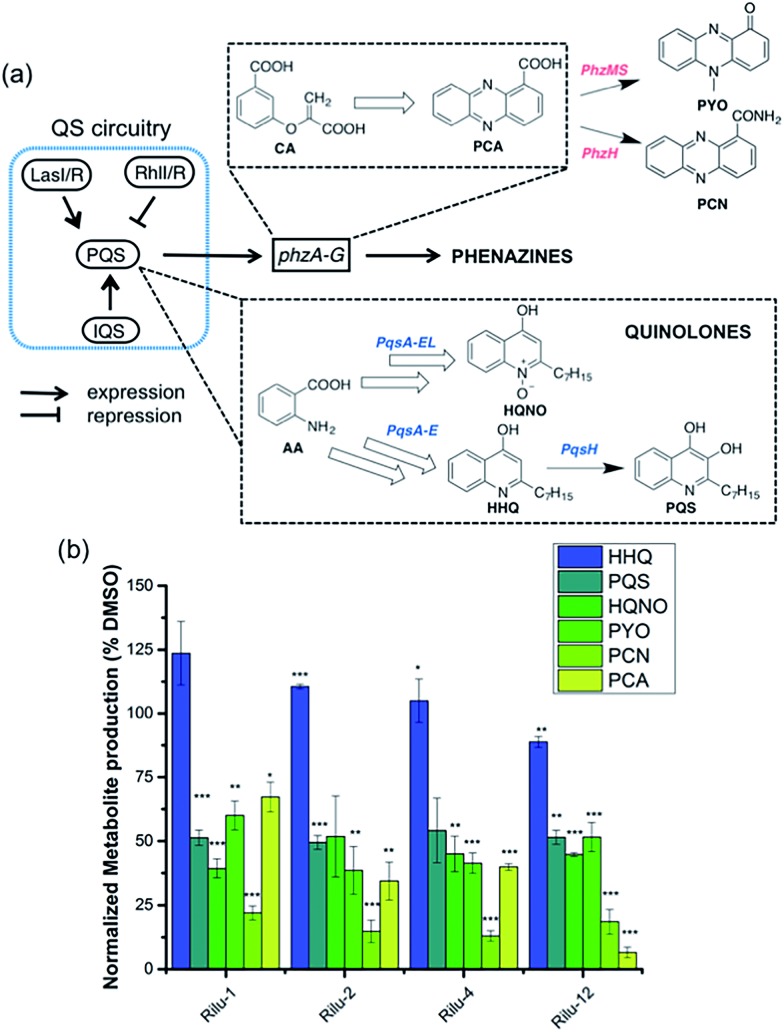
(a) Abbreviated model of the biosynthetic pathways for production of virulence-related metabolites and their relation to the QS network. (b) Effects of **Rilu**-compounds at 200 μM on the production of metabolites in PA14 after 9 h of incubation at 37 °C and 200 rpm. All values are plotted relative to a DMSO-treated control and normalized to the final cell density (OD_620_). Error bars represent the standard error of three independent experiments. Statistical analysis performed with non-parametric one-way ANOVA (*α* = 0.05; ***p < 0.001; **p < 0.02, *p < 0.05).

To determine if the HK inhibitors affect the pathogenicity of *P. aeruginosa*, we examined its production of two major classes of molecules involved in signaling and infection establishment, the *Pseudomonas* quinolone signals (**PQS**s) and the phenazines. At the TCS level, pathways such as GacS/GacA-RetS and PhoR/PhoB are associated with the formation of **PQS**s and phenazine metabolites *via* the QS system and small RNAs machinery (Fig. 2).^17^ However, a complete picture of the roles that the TCSs play in QS regulation is not yet available.^13^,^17^,^18^ Looking deeper into the QS network, production of the QS signals is tightly regulated by several circuits (Fig. 3a).^18^ We were interested in the **PQS** system as it autoregulates the production of quinolone-type compounds, which have roles in controlling other toxins and virulence behavior in *Pseudomonas*. When a quorum is reached, biosynthesis of 4-hydroxy-2-heptylquinoline (**HHQ**) and 3,4-dihydroxy-2-heptylquinoline (**PQS**) is initiated by the production of their precursor, anthranilic acid (**AA**).^18^,^19^ In another pathway, **AA** can also be converted to 4-hydroxy-2-heptylquinoline-N-oxide (**HQNO**). The release of **PQS** activates the phenazine-producing genes *phzA-G* that convert chorismic acid (**CA**) to the primary phenazine metabolite, phenazine-1-carboxylic acid (**PCA**; Fig. 3a).^17^ From different cues, **PCA** can be modified to phenazine-1-carboxamide (**PCN**) and to the other crucial toxin of *P. aeruginosa*, pyocyanin (**PYO**; Fig. 3a).^17^ Although not direct, it is evident that the expulsion of these toxic metabolites (quinolones, phenazines) is regulated by TCSs and as such, inhibiting the production of these toxins is a viable antibacterial therapy.^20^

To measure these metabolites, we carried out LC-MS/MS analyses on the supernatant of PA14 cultures grown in the presence or absence of inhibitors at various growth phases (OD_620_ = 0.9 in Fig. 3b, OD_620_ = 1.8 in Fig. S6, mass spectrometry data in Fig. S7†).^19^,^21^ Although the levels of **HHQ** were slightly increased upon treatment (**Rilu-1**, ∼24%), **PQS** production was markedly decreased (∼50%) by all inhibitors compared to the DMSO control (Fig. 3b). This result is quite exciting as null mutation of the *pqs* system affects other virulence factors such as **PYO**, elastase, and rhamnolipids (RLs).^18^ All of the inhibitors also significantly reduced the levels of **HQNO** by up to ∼50%. This is an important finding as **HQNO**, unlike QS-activity related quinolones,^22^ is an extremely crucial toxin for *P. aeruginosa* as it enables the bacteria to survive in hosts by aiding in extracellular DNA production and killing of other microbiota.^18^ In addition, up to a 70% reduction of **PYO** was achieved (**Rilu-2**), while **PCN** was reduced by ∼85% (**Rilu-2**) and finally, only negligible amounts of **PCA** (∼8%) were produced with **Rilu-12** (Fig. 3b). These results were corroborated by dramatic reduction in the characteristic green hue of *P. aeruginosa* cultures, which is due to multiple metabolites, including the phenazines and the siderophore pyoverdine (24 h inhibitor treatment; Fig. S6†). The turbidity of these cultures further validates that the compounds do not impede bacterial growth and that interfering with the aforementioned TCS machinery component(s) likely causes the striking reductions in toxin production.

**Fig. 4:**
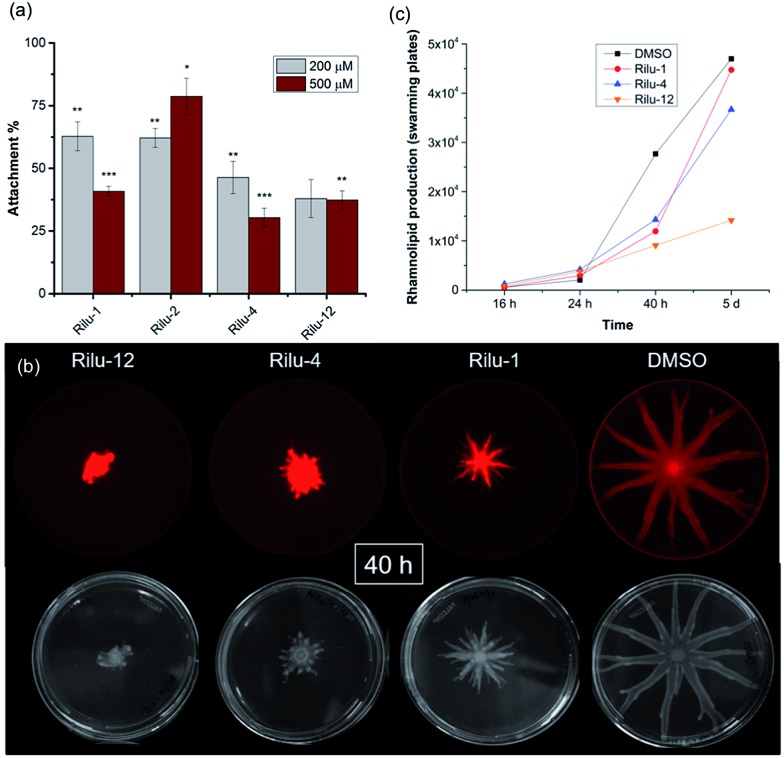
(a) Rapid attachment assay of PA14 in the presence of **Rilu**-compounds. All values are plotted relative to a DMSO-treated control and normalized to the final cell density (OD_620_). (b) Swarming motility of PA14 in the presence of **Rilu**-inhibitors after 40 h. The top images are fluorescence scans for detecting Nile red and the bottom images were taken with a digital camera. (c) Production of RLs in PA14 cultures on agar plates as quantified with ImageJ. Note: **Rilu-2** was omitted, as large quantities are needed for the swarming agar plates. Error bars represent the standard error of three independent experiments (*n* = 3). Statistical analysis performed with non-parametric one-way ANOVA (*α* = 0.05; ***p < 0.001; **p < 0.02, *p < 0.05).

We next sought to assess the ability of our molecules to interfere with *P. aeruginosa* virulence mechanisms. Biofilm formation (Fig. S8†) is indisputably the best way for a microorganism to establish its cellular community and in *P. aeruginosa*, a complex web of many TCSs controls biofilm formation. For example, TCSs GacS/GacA, PhoQ/PhoP, and NarL/NarX positively regulate biofilm formation, but when sensor kinase PA4398 was mutated in PA14, a 1.8 fold increase in biofilm production was seen (Fig. 2).^13^ Using a microtitre assay, we evaluated biofilm formation of PA14 cultures and observed 25% and 40% reduction of biofilm mass with high concentrations of **Rilu-2** and **4**, respectively (Fig. S8†). Although the leads caused only a modest reduction in biofilm formation, deeper examination of these microbial films can provide insights into the organism's ability to attach to solid surfaces, as alterations in appendages like flagella and type IV pili can result in dramatic architectural changes. Accordingly, an important corollary analysis is assessment of the initial surface adhesion of the pathogen (Fig. 4a) that is dependent on flagella/pili formation, with a “rapid attachment assay”.^8^,^23^ A notable difference in surface attachment was observed with all of the **Rilu**-compounds. In particular, a 70% reduction occurred in cultures containing **Rilu-4** or **12** (Fig. 4a).

Activation of HKs, such as chemotaxis-related WspE, ChpA and the nitrogen acquisition-related, PilS, has been linked to flagella and pili-synthesis and the pronounced effects of our molecules in the attachment assay implied that the **Rilu**-compounds may be affecting the motility of *P. aeruginosa. Pseudomonas* is unique in its application of versatile motility modes and among these, swarming enables the deadly pathogen to move through mucosal layers in CF patients based on nutritional and viscosity variations.^24^,^25^ The coordinated movement of bacteria using both flagella and type IV pili exhibit a greater resistance to multiple antibiotics and express higher levels of virulence-related factors compared to planktonic cultures.^24^ Swarming is TCS-dependent, as HKs *gacS*, and *fleS*, as well as alginate RR *algR* mutants are all impaired in swarming motility.^13^,^24^ We investigated if our inhibitors modify swarming behavior, as this is essential for pathogenicity. The **Rilu**-compounds (200 μM) dramatically affected swarming growth.^26^ After 40 h, a marked reduction in tendril formation is clearly observed with all compounds, most prominently with **Rilu-12** (Fig. 4b, bottom images). Even after 5 days, a moderate difference in swarming can be noted with **Rilu-1**, **4**, and a major impairment in motility is still visible with **Rilu-12** (Fig. S9†). A similar reduction in swarming was also seen with 125 μM of **Rilu-4**, **12** (Fig. S10†), indicating the efficacy of the compounds at lower concentrations.

To reduce surface tension and swarm adequately, *P. aeruginosa* cells secrete RLs, amphiphilic glycolipids.^25^ We utilized a fluorescent lipophilic stain, Nile red that can bind to these glycolipids, and visualized the swarming growth of PA14.^25^,^27^ Similar to the phase-contrast images, the stain is concentrated at the center of the swarm colony for all of the samples at 40 h (Fig. 4b, top images). Whereas in the 5 days images, the RLs are evenly distributed throughout the swarm area (Fig. S9†). We quantified the RL levels at 16 h, 24 h, 40 h and 5 days (Fig. 4c, S9 and S11†) and found that RL production is lowest in the presence of **Rilu-12** (70% reduction). The loss of swarming behavior provides ample evidence that the benzothiazole compounds heavily affect motility machinery and surfactant production.

**Fig. 5:**
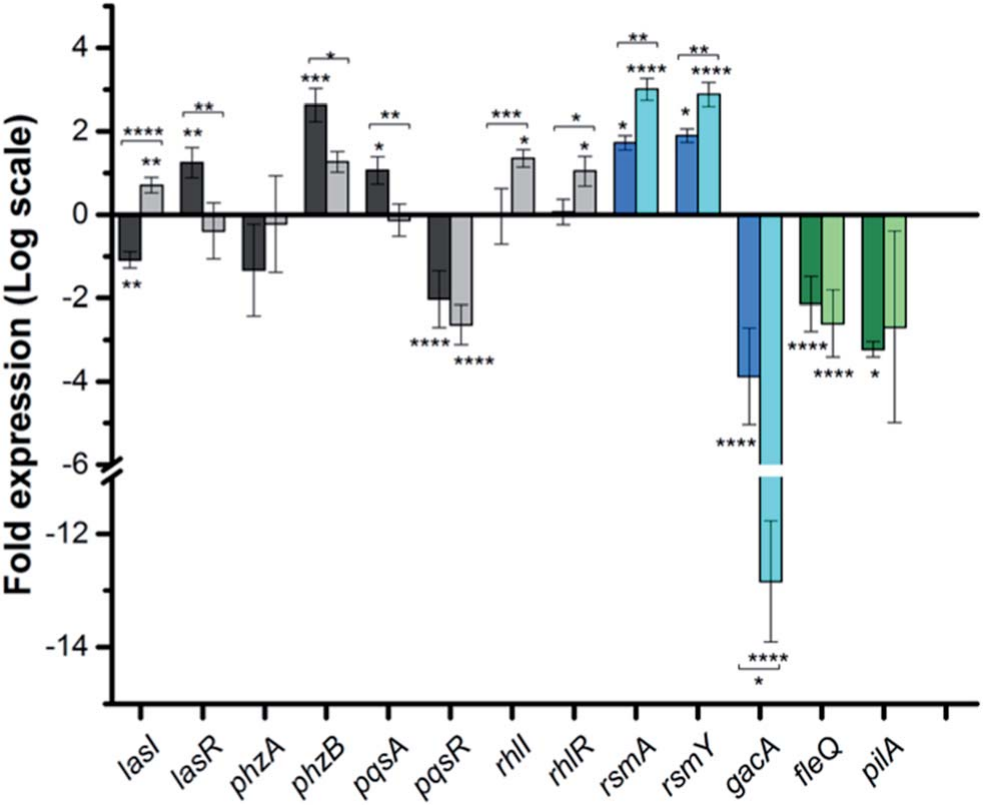
qPCR analysis of various genes in PA14 upon exposure to **Rilu-12**, fold expression is shown in log 2 scale. Genes are colored based on three classes: QS-related (grey), TCS GacSA-related (blue) and motility-related (green). Darker and lighter bars (grey, blue and green) are measurements for **Rilu-12** at 200 μM and 500 μM, respectively. Error bars represent the standard error of three independent experiments (*n* = 3). Statistical analysis performed with non-parametric one-way ANOVA (*α* = 0.05; ****p < 0.0001; ***p < 0.001; **p < 0.02, *p < 0.05). The asterisks closest to the bars denote the significance of the difference between the inhibitor-treated samples and the DMSO-treated sample. The asterisks outside of the brackets denote the significance of the difference between the two inhibitor concentrations.

To dig deeper into our TCS regulation postulation and to quantify genetic level changes, we performed RT-qPCR experiments. We examined the transcript levels of 14 genes that are linked to the pathogenicity, as well as the TCS machinery of *P. aeruginosa*. Two inhibitor concentrations (200 and 500 μM) were chosen to examine if transcription levels change in a dose-dependent manner and PA14 cultures from the OD_620_ experiments were used for RNA isolation for consistent results. We selected 8 genes that are QS signal-related and indirectly govern swarming and biofilm formation (colored in grey bars; Fig. 5 and S12†).^28^,^29^ *lasI*, *lasR* and *rhlI*, *rhlR* encode the synthases and receptors of the QS systems, Las and Rhl, respectively. *pqsA*, *pqsR* govern **PQS** synthesis and response and *phzA*, *phzB* are involved in phenazine biosynthesis.^30^ For many of these genes namely, *lasI*, *lasR*, *rhlI*, *rhlR*, *pqsA* and *phzA*, only small transcriptional changes were observed with the **Rilu**-compounds (Fig. 5 and S12†). This is at odds with the substantial decrease in production of RLs in the swarm plates that we noted, which is dictated by *rhl*. This discrepancy is likely due to the fact that the microorganism requires more glycolipids to move/colonize on solid surfaces, while it does not need much in liquid media. To investigate this, we quantified RLs in treated PA14 liquid cultures using a phenol-orcinol assay and found no difference in the levels of glycolipids, corroborating the qPCR results (Fig. S13†). This observation indicated that transcription levels could vary greatly in *Pseudomonas* from liquid cultures to swarm semi-solid cultures. In fact, Deziel *et al.* reported vast differences in gene expression between the center and tendril tips of a *P. aeruginosa* swarm colony.^31^ On the other hand, the **PQS** response gene (*pqsR*), also known as multi-virulence factor (*mvfR*), showed a ∼3-fold decrease in expression with all of the **Rilu**-inhibitors.^18^ This regulation is quite significant as *pqsR*, the cognate receptor and co-inducer of **PQS**, is essential for executing **PQS** signal transduction, and controls synthesis of the phenazines. Thus, the lower expression of *pqsR* is consistent with the reduced metabolite production, such as **PQS** and **HQNO**, seen in Fig. 3b. This may affect other phenazine biosynthetic enzymes (PhzC-G or PhzMS/H) as is suggested by the dramatically altered **PCA**/**PCN**/**PYO** levels (Fig. 3b).^17^,^18^

Next, we examined *gacA*, the RR of the super-regulator TCS GacA/GacS whose expression directly upregulates expression of the small regulatory RNA *RsmY*, which captures the small RNA-binding regulatory protein RsmA (Fig. 2; encoded by *rsmA*, also quantified).^32^ All of these genes are colored as blue bars in Fig. 5 and S12.† The expression of *gacA* was dramatically reduced (up to ∼15 fold), suggesting that the sensor kinase, GacS is inhibited, which controls its cognate RR GacA levels. In turn, we expected to see reduction of *RsmY*, but observed the opposite trend. This outcome possibly stems from the fact that *RsmY* regulation is complex and while GacS positively regulates *RsmY*, four other HKs, namely BfiS (not shown in Fig. 2), PA1611, PA1976, and SagS repress it through the histidine phosphotransfer protein, HptB (Fig. 2).^33^ Similarly, *rsmA* transcription is not only controlled by *RsmY* but another small regulatory RNA, *RsmZ* influences its expression.^33^ We noted a slight up-regulation of *rsmA* that potentially has effects on the Las, Rhl systems. While *gacA* reduction is promising and supports our HK-inhibition hypothesis, full transcriptome analysis along with affinity probe-pull down experiments will be needed to provide conclusive evidence for the TCS inhibition events that are most critical to the observed phenotypic results. Finally, since we hypothesized that the reduction in swarming and surface attachment is related to motility mechanisms, we investigated the transcript levels of *fleQ* (flagellar regulator) and *pilA* (type IV pili fimbrial precursor).^24^,^25^,^34^ Both are colored as green bars in Fig. 5 and S12.† These genes are heavily regulated by chemotaxis-related TCSs and HKs involved with cyclic-di-GMP messenger levels. In fact, the Sintim lab recently demonstrated that a cyclic-di-GMP esterase inhibitor can interfere with swarming and RL production in PAO1.^35^ Other reports have connected mutants of *fleQ* and *pilA* with deficiencies in swarming and twitching motility.^24^,^25^,^34^ Gratifyingly, expression of both of these genes was decreased by up to ∼3.5 fold, which corroborates the significant defects observed in the swarming and surface attachment experiments.

Finally, to provide further evidence that the *in vitro* activity of the HK inhibitors is correlated with what is observed in the whole cell assays, we performed transcription analysis with a **Rilu**-analog that was inactive for HK inhibition, **Rilu-7**. We observed that this compound does not significantly affect the expression of virulence-associated genes (*pqsR*, *fleQ* and *pilA*), which were substantially downregulated by our lead molecules, **Rilu-4** and **12** (Fig. S12†). We also found that although **Rilu-4** and **12** caused a dramatic downregulation (12–15 fold) of *gacA*, the “super regulator” of virulence, the expression of this gene was essentially unchanged in the presence of **Rilu-7** (1.2 fold change). These promising results further strengthen our hypothesis that the antivirulence activity that we observe with the **Rilu**-leads is due to inhibition of crucial HK targets and subsequent signal transduction in bacteria.

## Conclusions

*P. aeruginosa* is a versatile organism and has an extremely intricate virulence network that is tightly controlled by TCSs.^36^ We have shown that by blocking TCS signaling, a remarkable attenuation in virulence behavior can be achieved. Inhibition of HKs, the kinase partner in TCSs, was carried out with benzothiazole-containing compounds. Among these, **Rilu-4** and **12** are the most promising as they significantly reduce the production of virulence factors such as **PQS**, toxins like **PYO**, **PCN**, and **PCA**, and severely impact the motility behavior of PA14. Transcription analyses showcased the regulation of a crucial TCS GacS/GacA that is essential to *P. aeruginosa*'s pathogenicity and provides evidence of other TCS signaling inhibition. Current efforts in our lab are aimed at improving the efficacy of the **Rilu**-inhibitors, understanding the key inhibition mechanisms, and evaluating the identified molecules in additional drug-resistant strains of *P. aeruginosa*.

## Conflicts of interest

There are no conflicts to declare.

## Supplementary Material

Supplementary information
