# Supplementary material for: Disarming the virulence arsenal of Pseudomonas aeruginosa by blocking two-component system signaling
Source: Chem Sci. 2018 Jul 10;9(37):7332–7. doi: 10.1039/c8sc02496k (PMC6237130; doi:10.1039/c8sc02496k)
Supplement: Supplementary file 1 [file SC-009-C8SC02496K-s001.pdf]

Electronic Supplementary Material (ESI) for Chemical Science.
This journal is © The Royal Society of Chemistry 2018

Supporting Information

Disarming the virulence arsenal of *Pseudomonas aeruginosa* by blocking two-component system signaling

Manibarsha Goswami,[1] Adeline Espinasse,[1] and Erin E. Carlson[1,2,3]*

[1]Department of Chemistry, University of Minnesota, 225 Pleasant St. SE, Minneapolis, MN, 55454, United States

[2]Department of Medicinal Chemistry, University of Minnesota

[3]Department of Biochemistry, Molecular Biology and Biophysics, University of Minnesota

Contents **Page No.**

Materials and Methods

Protein Production of WT HK853…………………………………………….. S2-S3
Inhibition activity results of inhibitors………………………………………. S4-S9
Aggregation analysis of selected inhibitors………………………………… S11
Growth of PA14 cultures with inhibitors……………………………………. S12
HSP90 inhibition assay with inhibitors…………………………………….. S13
LC-MS analyses of metabolites……………………………………………. S14-S17
Biofilm and attachment assays……………………………………………..... S18-S19
Swarming assays and rhamnolipids quantification……………………….. S20-S21
RT-qPCR experiments………………………………………………………. S22-S23
Liquid cultures rhamnolipids production……………………………………. S24
References…………………………………………………………………. S25

MATERIALS AND METHODS

Protein Production

HK853 overexpression and purification. HK853 in the pHis-parallel vector was prepared as described previously.[1] DNA was transformed into competent BL21(DE3)-pLysS Rosetta *E. coli* cells. Transformed *E. coli* cells were plated overnight on lysogeny broth (LB) agar containing 100 µg mL^{-1} ampicillin (amp) and 34 µg mL^{-1} chloramphenicol (Cm). A single colony was transferred to 100 mL sterile LB media in a 250-mL flask supplemented with antibiotics and incubated at 37 °C overnight at 220 rpm. At OD_{620} of 0.4–0.6, 15 mL was transferred to 1 L sterile LB broth containing antibiotics in 2.8-L baffled flasks. Cultures were grown by shaking at 220 rpm at 37 °C to an OD_{600} ~0.6. After equilibrating to 20 °C for 3 h, HK853 overexpression was induced with 0.22 mM of isopropyl β-D-1-thiogalactopyranoside (IPTG; Calbiochem) and incubation at 20 °C for 16 h at 220 rpm. Cells were collected by centrifugation at 8000 *xg* for 20 min, resuspended in 10 mL of buffer (25 mM Tris-HCl, pH 8, 500 mM NaCl, 10% glycerol, and 2 mM DTT), and quickly frozen on dry ice for storage at –80 °C.

For purification, each pellet from 1 L of culture was resuspended in a total volume of ~50 mL lysis buffer (25 mM Tris-HCl, pH 8, 500 mM NaCl, 10% glycerol, and 2 mM DTT) containing 20 units Deoxyribonuclease I (Sigma) and four Complete Mini EDTA-free protease inhibitor tablets (Roche). Resuspended cells were lysed by a Branson Sonifier 250 with 1/8-inch tapered microtip (power setting 3.5, duty cycle 30%) for 1 h 20 min on ice. Lysate was centrifuged at 14,000 x*g* for 40 min at 4 °C. The supernatant was collected and filtered (0.22 µm). Using an ÄKTApurifier (GE Healthcare) at 4 °C, HK853 was purified from lysate by nickel affinity on a nickel-nitriloacetic acid column (Ni-NTA; Qiagen). Ni-NTA buffer was 25 mM Tris-HCl, pH 8, 500 mM NaCl, 10% glycerol, and 2 mM DTT. An elution gradient of 5 mM imidazole (buffer A) to 1 M imidazole (buffer B) was used to elute His-tagged protein. Eluted HK853 was concentrated for size exclusion chromatography on a HiLoad 16/600 Superdex 75 pg column (GE Healthcare) using 10 mM Tris-HCl, pH 7.6, 0.1 mM EDTA, 0.5 M NaCl, 12% glycerol, and 2 mM DTT. This buffer was also used for storage of protein at –80 °C, in which protein was flash frozen on dry ice/isopropanol. Protein concentration was determined using the DC Protein Assay (Bio-Rad).

Reaction buffer. Used in all assays, the reaction buffer was composed of 50 mM Tris-HCl, pH 7.8, 200 mM KCl, 5 mM $MgCl_2$.

Protein Storage Buffer. Buffer for the storage of protein was prepared as 10 mM Tris-HCl, pH 8, 0.1 mM EDTA, 0.5 M NaCl, 12% glycerol, 2 mM DTT.

Determination of protein concentration. Protein stock concentrations were determined by a DC Protein Assay (Bio-Rad) according to the instruction manual and with BSA as a standard. The concentrations of at least two dilutions of protein stock were determined and averaged. Where indicated, protein concentration was also determined using an Implen's Nanophotometer spectrophotometer (Thermo Fisher Scientific) at 280 nm and Beer's Law,

$$A = \varepsilon c \ell \qquad \text{(Equation 1)}$$

where A is absorbance, ε is the protein extinction coefficient ($M^{-1}cm^{-1}$), c is concentration (M), and ℓ is pathlength (cm).

SDS-PAGE. 2X SDS-PAGE sample loading buffer contained 125 mM Tris, pH 6.8, 20% glycerol, 4% SDS (w/v), 5% 2-mercaptoethanol, and 0.2% bromophenol blue (w/v). Tris-glycine stacking gels were prepared with a 10% polyacrylamide resolving gel and 4.5% polyacrylamide stacking gel. Running parameters were 180 V, 400 mA, and 60 W for 1 h 20 min. SDS-PAGE running buffer was diluted ten-fold from Novex 10X Tris-Glycine SDS Running buffer (Invitrogen) and pre-chilled prior to electrophoresis.

Native-polyacrylamide gel electrophoresis (Native-PAGE). Native-PAGE sample loading buffer contained 40 mM Tris, pH 7.5, 8% glycerol, and 0.08% Bromophenol blue (w/v). Native-PAGE gels were 7.5% polyacrylamide Tris-glycine resolving gels. Running parameters were 180 V, 400 mA, and 60 W for 1 h 20 min. The pre-chilled electrophoresis running buffer was 83 mM Tris, pH 9.4, and 33 mM glycine.

Gel fluorescence detection. After SDS-PAGE, gels were washed three times with MQ water. They were scanned on a Typhoon Variable Mode Imager 9500 (GE) using 526-nm (short-pass filter) detection for BODIPY (λex: 504 nm, λem: 514 nm).

Coomassie staining. Each step was carried out at room temperature (RT) with an orbital shaker. After SDS-PAGE, gels were washed three times with MQ water and submerged in enough coomassie stain (0.1% (w/v) Coomassie Brilliant Blue R-250, 10% acetic acid, 40% methanol) to cover the gel and incubated for 10 min. Stain was removed, and destain (10% acetic acid, 40% methanol) was added to gel and incubated 30 min. After removing destain, gel was washed in water overnight. After staining, they were scanned on a Typhoon Variable Mode Imager 9500 (GE) using the coomassie stain settings.

Silver staining. Native-PAGE gels were silver stained. All steps were carried out at RT with an orbital shaker. The gels were stained using a Pierce™ Silver Stain Kit (ThermoFisher Scientific) following manufacture's protocol. After staining/destaining, they were scanned on a Typhoon Variable Mode Imager 9500 (GE) using silver stain settings.

Inhibition of HK853 Activity

Commercially available probes (BODIPY-ATPγS) and inhibitors (**Rilu-1-12**) were obtained from Sigma-Aldrich, Thermo Fisher Scientific, Alfa Aesar and TCI America at >97% purity. Unless otherwise indicated, all commercially available chemicals were used without further purification.

BODIPY-ATPγS competition screening was performed at inhibitor concentrations that did not cause aggregation. Triton X-100 was premixed with reaction buffer to yield 0.1% (v/v) in final 25-μL reactions. In reaction buffer, 1 μM HK853 was preincubated with test compounds (final concentration, 0.01−1250 μM) in 24 μL for 30 min. 1 μL BODIPY-ATPγS was added to bring the final 25-μL reactions to 0.96 μM HK853 and 2 μM BODIPY-ATPγS in the presence of competitors and 5% DMSO. Samples were mixed and incubated in the dark at RT for 1 h before quenching with 8.6 μL 4× SDS-PAGE sample loading buffer and loading 15 μL on a 10% stacking gel. After SDS-PAGE, in-gel fluorescence detection elucidated HK853 activity, and coomassie staining of the gels ensured even protein loading. Integrated density values of the fluorescent gel bands were normalized as "% Activity" with respect to a control that contained no inhibitor. Data were plotted in GraphPad Prism with relation to the log of molar inhibitor to determine IC_{50} values (Equation 2).

Data analysis. Integrated density measurements of in-gel fluorescence and phosphorescence were performed in ImageJ.[2] Data were prepared and analyzed in GraphPad Prism (version 7.0 for Mac, GraphPad Software, San Diego, California USA, www.graphpad.com). For all DRCs (control FP competition and activity assays), data were fit to a four-parameter logistic equation,

$$y = Bottom + \frac{(Top - Bottom)}{1 + 10^{\left((LogIC_{50} - x) * HillSlope\right)}} \qquad \text{(Equation 2)}$$

where y is the response, *Bottom* and *Top* are plateaus in the units of the y-axis, x is the log of the molar concentration of inhibitor, *HillSlope* is the slope of the curve, and IC_{50} is the concentration of compound required for 50% inhibition (a response half way between *Bottom* and *Top*). Some compounds exhibited incomplete DRCs because going to higher concentrations would increase the required DMSO or cause protein aggregation. Visually, this meant there was no curve plateau for the "Bottom" value. However, IC_{50} values were desirable for purposes of comparison to other compounds. As a result, IC_{50} values were estimated by constraining the bottom of the curve to "0."

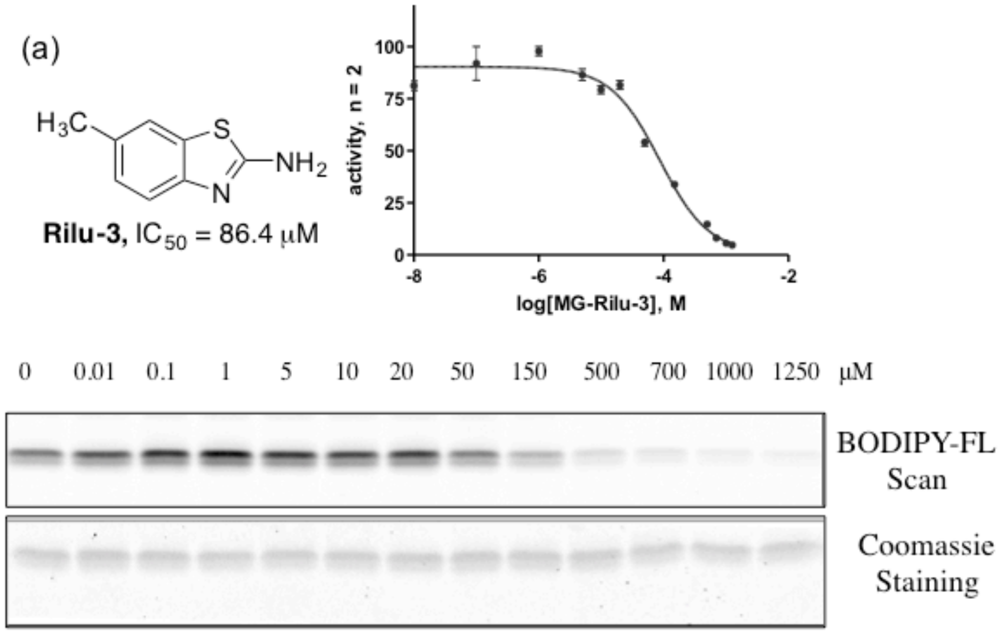
(a)
H3C
S
NH2
N
Rilu-3, IC50 = 86.4 μM
activity, n = 2
100
75
50
25
0
-8
-6
-4
-2
log[MG-Rilu-3], M
0 0.01 0.1 1 5 10 20 50 150 500 700 1000 1250 μM
BODIPY-FL Scan
Coomassie Staining

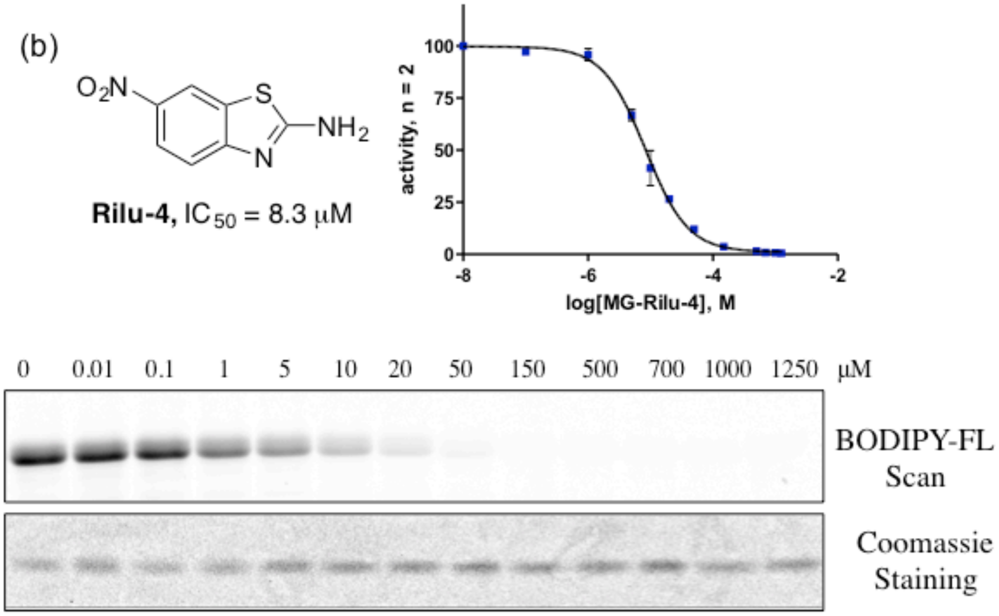
(b)
O2N
S
NH2
N
Rilu-4, IC50 = 8.3 μM
activity, n = 2
100
75
50
25
0
-8
-6
-4
-2
log[MG-Rilu-4], M
0 0.01 0.1 1 5 10 20 50 150 500 700 1000 1250 μM
BODIPY-FL Scan
Coomassie Staining

(c)

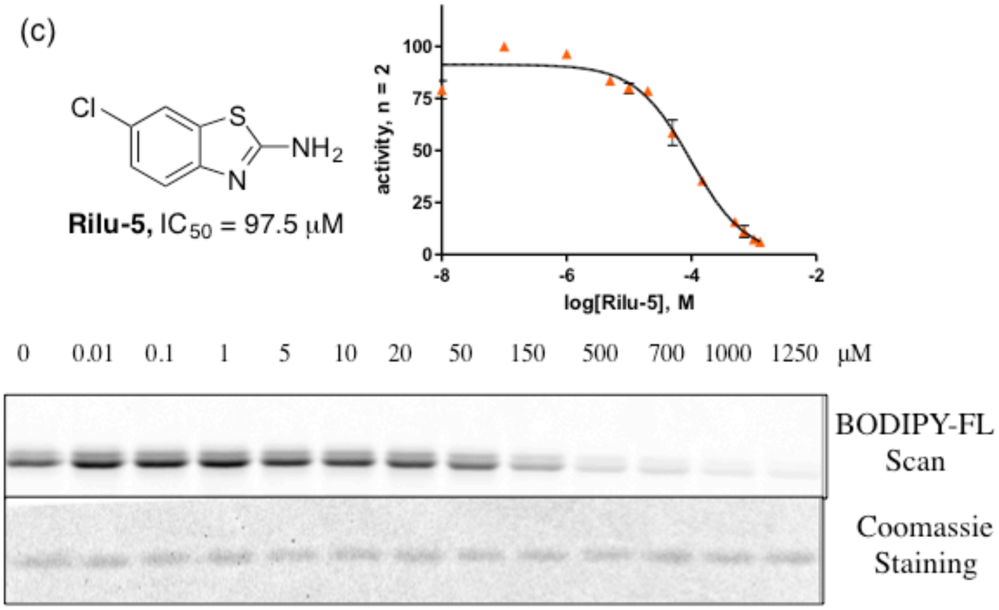
Cl
S
NH2
N
Rilu-5, IC50 = 97.5 μM
activity, n = 2
100
75
50
25
0
-8
-6
-4
-2
log[Rilu-5], M
0 0.01 0.1 1 5 10 20 50 150 500 700 1000 1250 μM
BODIPY-FL Scan
Coomassie Staining

(d)

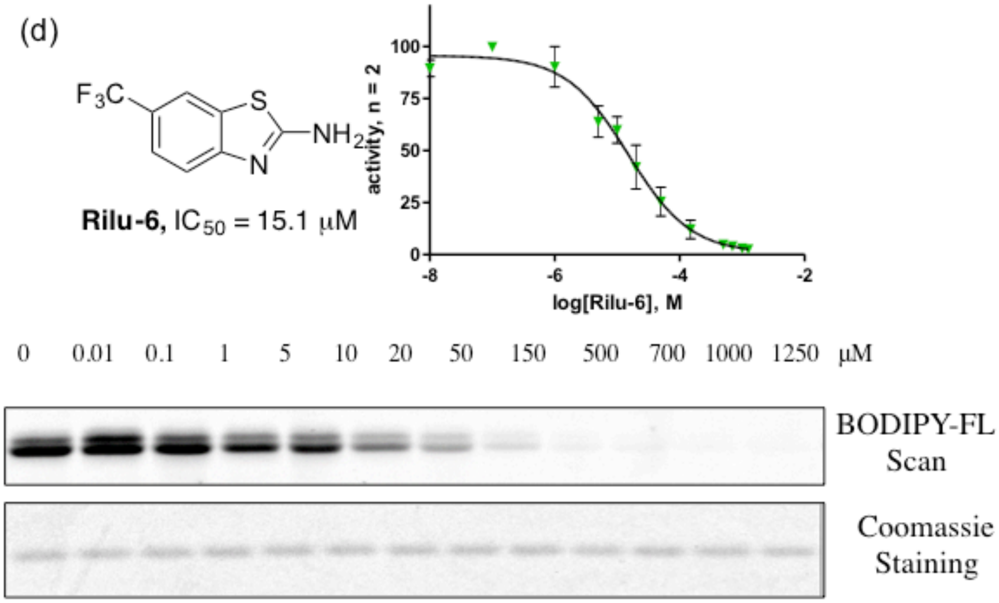
F3C
S
NH2
N
Rilu-6, IC50 = 15.1 μM
activity, n = 2
100
75
50
25
0
-8
-6
-4
-2
log[Rilu-6], M
0 0.01 0.1 1 5 10 20 50 150 500 700 1000 1250 μM
BODIPY-FL Scan
Coomassie Staining

(e)

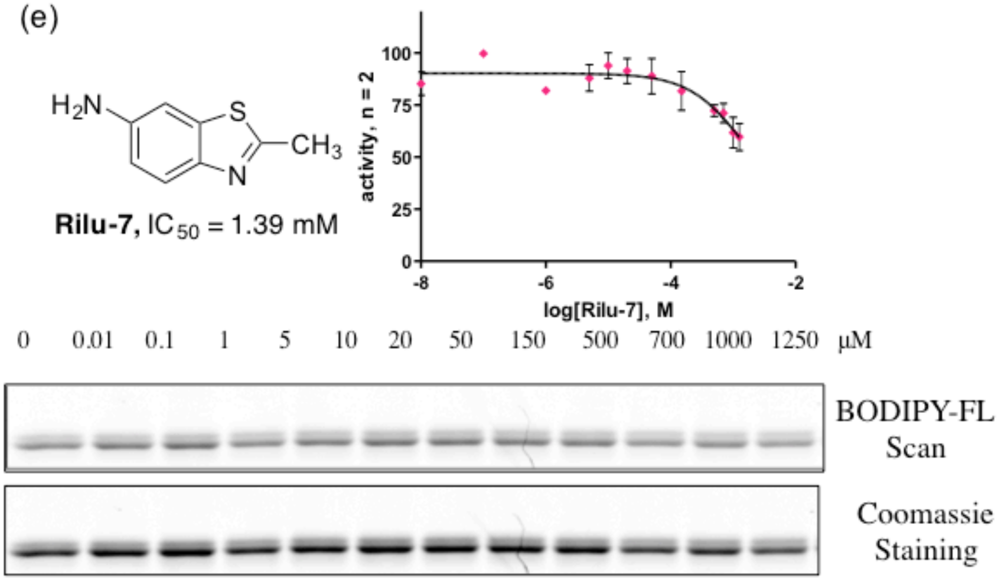
H2N
S
CH3
N
Rilu-7, IC50 = 1.39 mM
activity, n = 2
log[Rilu-7], M
0 0.01 0.1 1 5 10 20 50 150 500 700 1000 1250 μM
BODIPY-FL Scan
Coomassie Staining

(f)

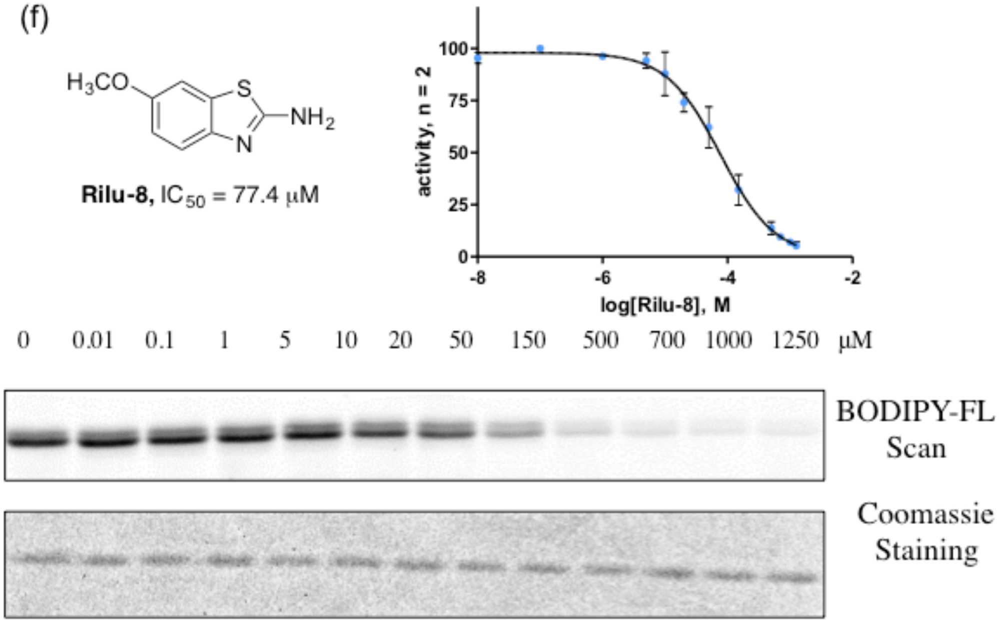
H3CO
S
NH2
N
Rilu-8, IC50 = 77.4 μM
activity, n = 2
log[Rilu-8], M
0 0.01 0.1 1 5 10 20 50 150 500 700 1000 1250 μM
BODIPY-FL Scan
Coomassie Staining

(g)

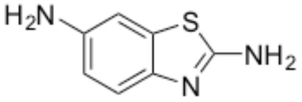

Rilu-9, IC_{50} = 161 μM

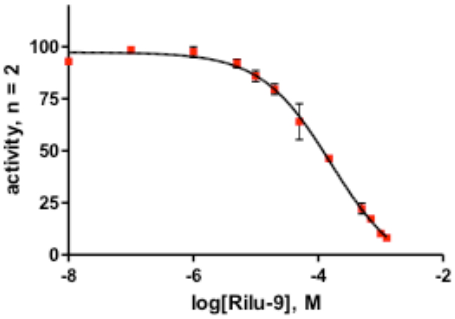

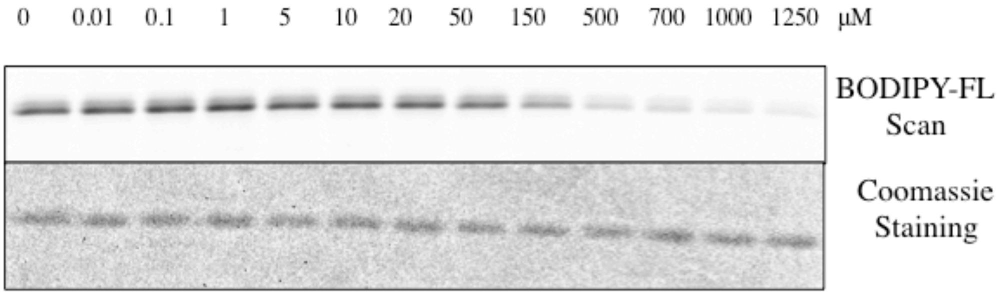

(h)

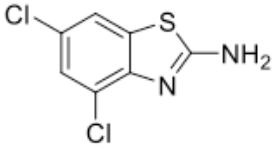

Rilu-10, No inhibition

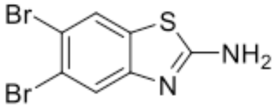

Rilu-11, No inhibition

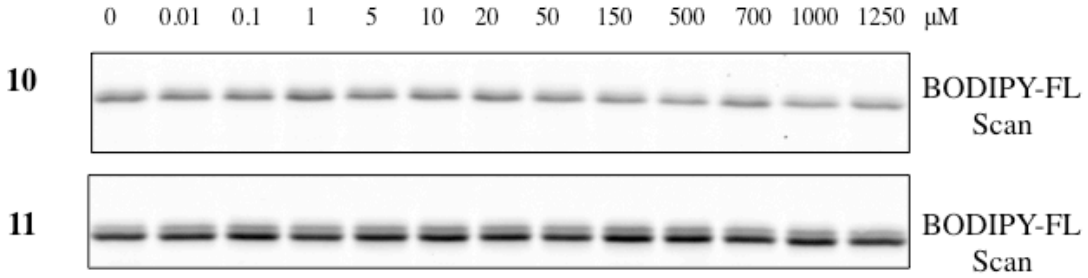

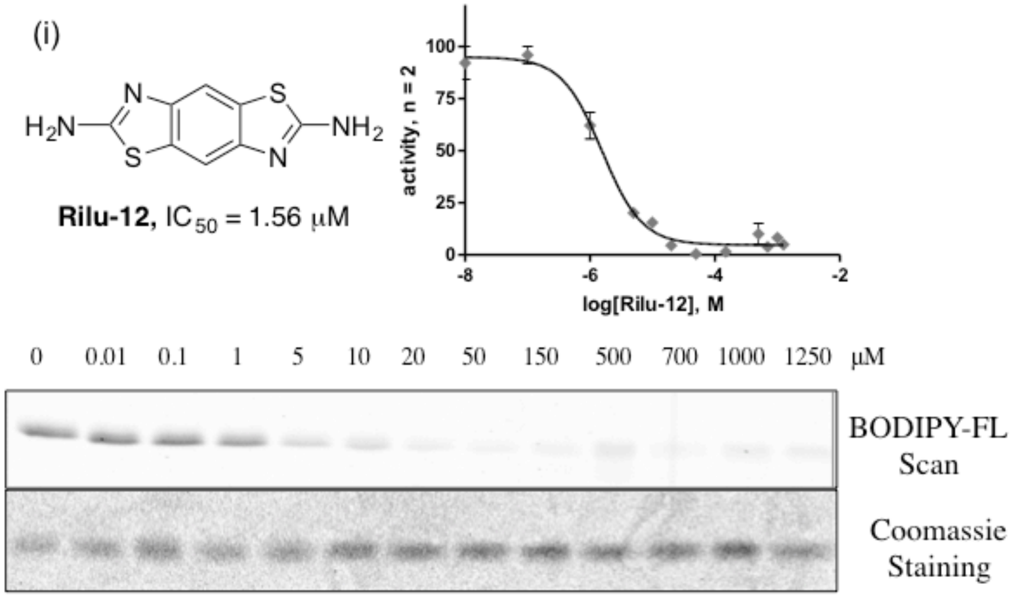

Figure S1: (a-i) Inhibition of HK853 activity with various **Rilu**-inhibitors.

Table S1. IC_{50} values of **Rilu**-compounds on HK853 inhibition.

Cmpd #	IC_{50} values (μM) (95% confidence interval), n = 2	Cmpd #	IC_{50} values (μM) (95% confidence interval), n = 2
**Rilu-3**	86.4 (5.81 to 128.3)	**Rilu-8**	77.4 (50.7 to 118.1)
**Rilu-4**	8.30 (7.27 to 9.47)	**Rilu-9**	161 (88.15 to 295.6)
**Rilu-5**	97.5 (57.5 to 165.2)	**Rilu-10**	No Inhibition
**Rilu-6**	15.1 (9.98 to 22.7)	**Rilu-11**	No Inhibition
**Rilu-7**	1394 (150 to 12940)	**Rilu-12**	1.56 (1.17 to 2.06)

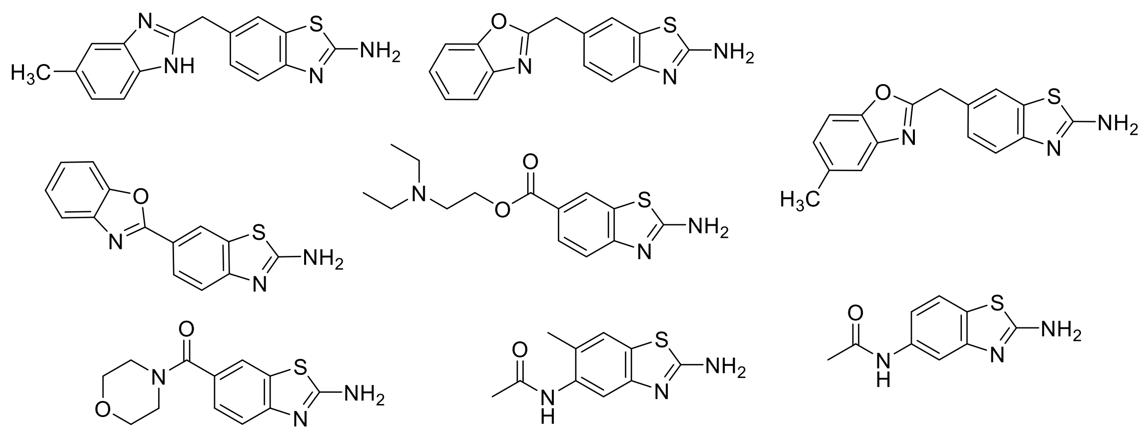

Figure S2. Structures of non-leads from the HTS that contain the benzothiazole scaffold. These compounds were found to be protein aggregators and were not pursued further (100 μM-1250 μM). [3]

HK853 Aggregation Analysis

To analyze the propensity for compounds to cause aggregation, each was mixed at six concentrations (0−1250 µM) with purified 0.5 µM HK853 in 25 µL of 20 mM HEPES buffer (5% (v/v) DMSO final). After incubating at RT for 30 min, 8.6 µL native-PAGE sample loading buffer was added, and 15 µL was loaded onto a 7.5% polyacrylamide gel. Proteins were resolved by native-PAGE and silver staining. Compound-induced aggregation was detected by the disappearance of the dimeric HK853 band. NH125 (Tocris Bioscience) was used as a positive aggregation control.

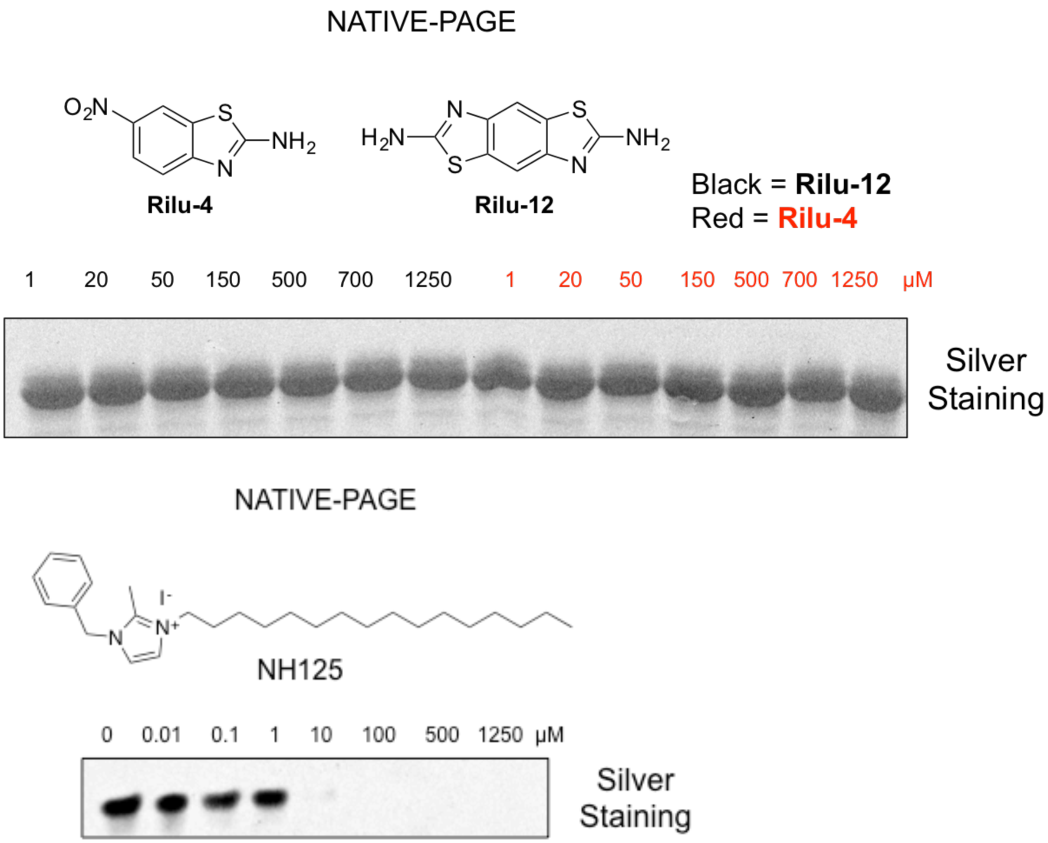

Figure S3: Native-PAGE of HK853 in presence of **Rilu-4**, **12** and known HK aggregator, NH125 (for comparison). Protein aggregation with NH-125 causes disappearance of the protein dimer band at ≥ 10 µM.

Growth of PA14 cultures with inhibitors

P. aeruginosa PA14 strain was grown overnight (~16 h) in LB media at 37 °C and 200 RPM. The overnight culture was diluted 1:100 in fresh LB media. 4 mL of this culture was then put into a 15 mL borosilicate glass tube, to which **Rilu**-inhibitors in DMSO were added to a final concentration of 200 μM and 500 μM (for the control experiments, the same amount of DMSO was added). These cultures were grown for 24 h at 37 °C and 200 RPM, and their OD_{620} values were measured using a spectrophotometer at various time points. These experiments were done in triplicate.

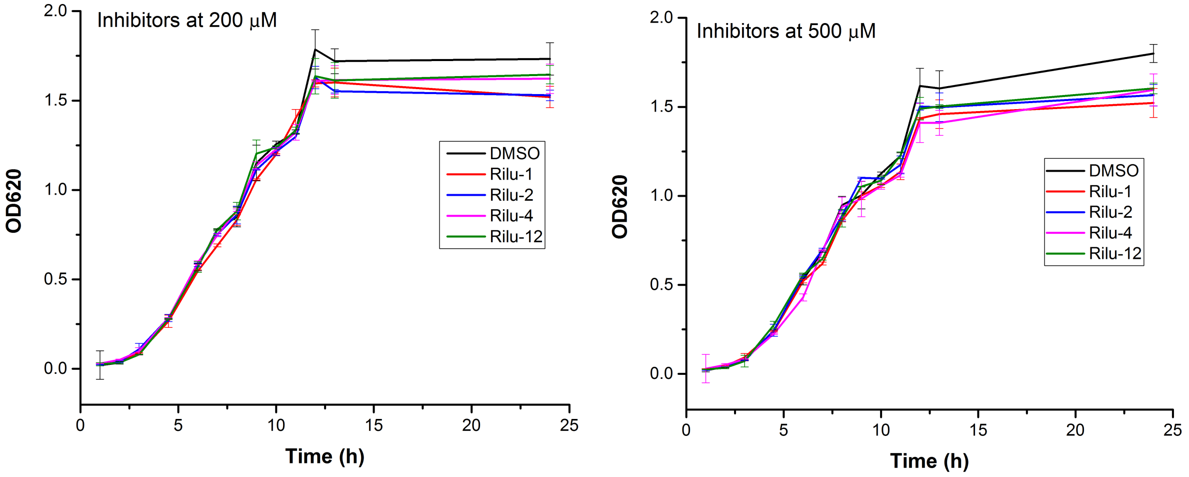

Figure S4: OD_{620} measurements of PA14 grown in the presence of different **Rilu**-compounds at 200 μM and 500 μM after 16 h at 37 °C. Error bars represent the standard error of three independent experiments (n = 3).

Heat-shock protein (HSP90) inhibition assay with inhibitors

Commercially available BPS Bioscience kit was used for HSP90α inhibition analysis at different concentrations of inhibitors (0.00001 – 1.25 mM) and in triplicates (n=3). For the positive control, geldanamycin, a known HSP90 inhibitor was used (IC_{50} ~50 nM).

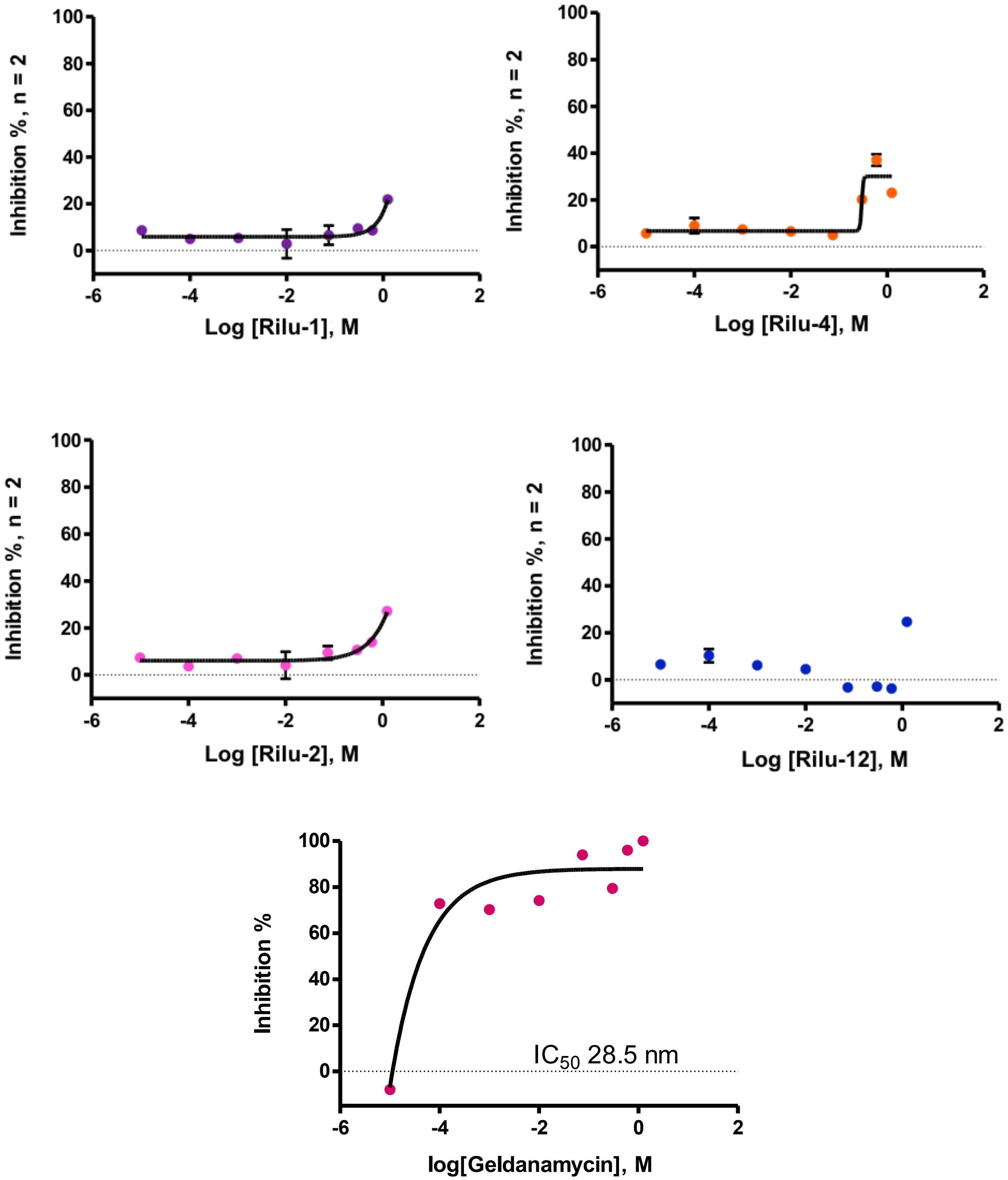

Figure S5. HSP90α inhibition assay results with various benzothiazole compounds and HSP90 inhibitor, Geldanamycin, and their dose-response curves.

LC-MS analyses of metabolites

P. aeruginosa PA14 strain was grown overnight (~16 h) in LB media at 37 °C and 200 RPM. The overnight culture was diluted 1:100 in fresh LB media. 4 mL of this culture was added into a 15 mL borosilicate glass tube, to which **Rilu**-inhibitors in DMSO were added to a final concentration of 200 μM (for the control experiments, same amount of DMSO was added). These cultures were grown for 9 h (late exponential phase) or 24 h (stationary phase) at 37 °C and 200 RPM. The OD_{620} values were measured using a spectrophotometer. 1 mL of each culture was placed in a 1.7 mL Eppendorf tube, centrifuged at 5,000 *g* for 5 min and the supernatant was removed from the cell pellets. The supernatants were directly analyzed on an ESI-LC-MS/MS instrument for metabolites and the cell pellets (from 9 h cultures) were further used for RNA extraction in q-PCR experiments.

ESI-LC-MS/MS analysis was performed on an Agilent 1290 Infinity/MS Agilent 6540 instrument. Injections of 2 μL were separated on Zorbax Eclipse plus C18 column (2.1 mm x 50 mm) and with mobile phases A (5% MeOH:95% water with 0.1% formic acid) and B (95% MeOH:5% water with 0.1% formic acid). The metabolites were separated using an isocratic elution of 100% for 0-3 min, then a 3-17 min linear gradient to 100% B, and 17-20 min of 100% B at a constant flow rate of 0.4 ml/min. Samples were measured in positive ion mode and data was collected in triplicate. Data were analyzed using the Agilent MassHunter Quantitative Analysis suite. From the extracted ion chromatograms (EICs) of the respective metabolite signals, the peak areas were normalized to the OD_{620} growth values of the respective cultures. All of the peak areas of the treated samples were plotted by considering the DMSO-control sample to be 100% abundance. The following ions were analyzed:

Table S2. Metabolites examined using LC-MS. Elution times and mass data are provided.

Metabolite	Retention Time (RT, min)	Expected $[M+H]^+$	Observed $[M+H]^+$	PPM difference
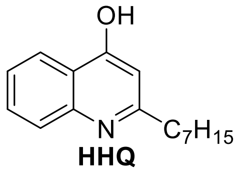 **HHQ**	12.8-13.1	244.1696	244.1684	-4.9
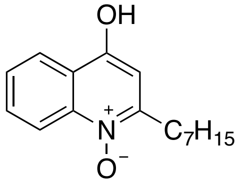 **HQNO**	13.3-13.7	260.1645	260.1628	-6.5

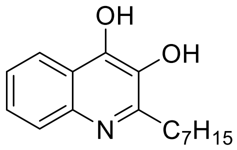 PQS	13.8-14.0	260.1645	260.1630	-5.7
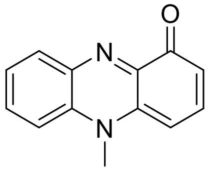 PYO	5.15-5.30	211.0866	211.0855	-5.2
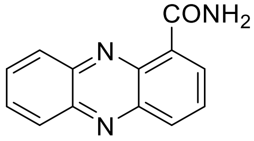 PCN	10.30-10.45	224.0818	224.0809	-4.2
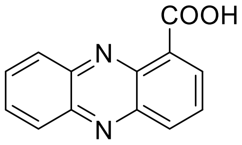 PCA	10.57-10.75	225.0659	225.0645	-6.6

Table S3. OD_{620} values for metabolomics

Compound	t = 9 h, OD_{620}	t = 24 h, OD_{620}
**DMSO**	0.860	1.72
**Rilu-1**	0.817	1.63
**Rilu-2**	0.851	1.60
**Rilu-4**	0.842	1.65
**Rilu-12**	0.883	1.65

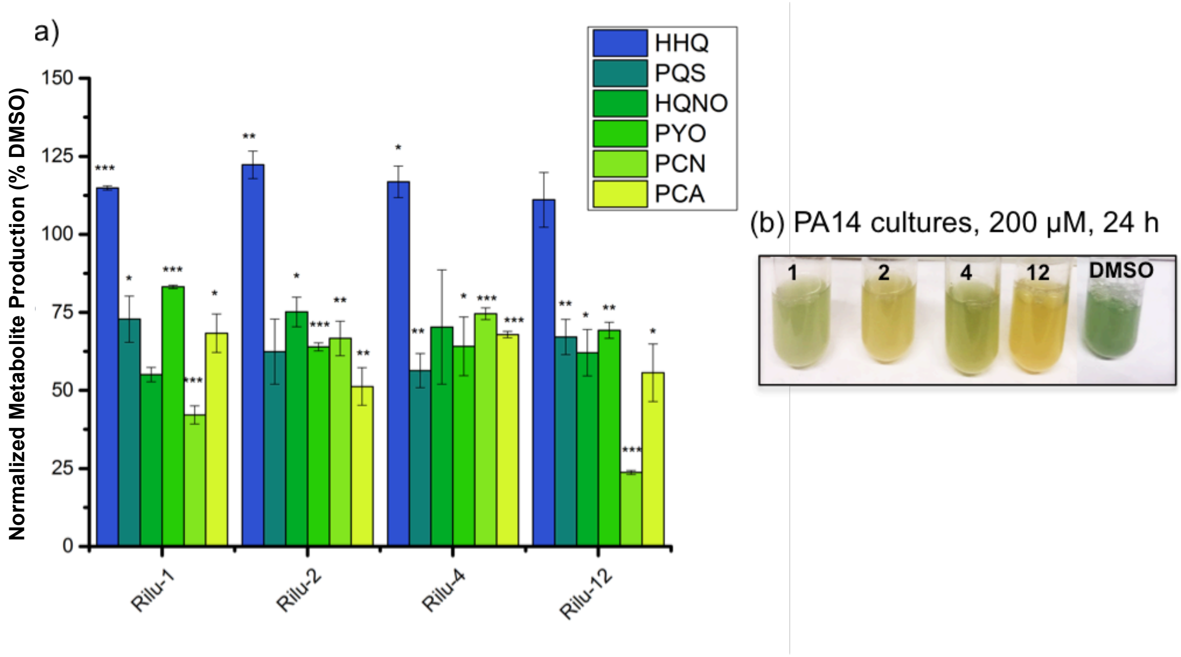

Figure S6. (a) Effects of **Rilu**-compounds at 200 μM on the production of metabolites in PA14 after 24 h of incubation at 37 °C and 200 RPM. All values are plotted relative to a DMSO-treated control and normalized to the final cell density (OD_{620}). Error bars represent the standard error of three independent experiments. Statistical analysis performed with non-parametric one-way ANOVA ($\alpha = 0.05$; ***$p < 0.001$; **$p < 0.02$, *$p < 0.05$). (b) PA14 cultures after 24 h (OD_{620} ~1.8) in presence of **Rilu**- compounds and DMSO at 200 μM.

a.

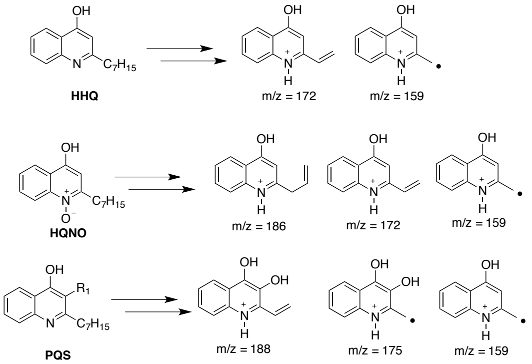

b.

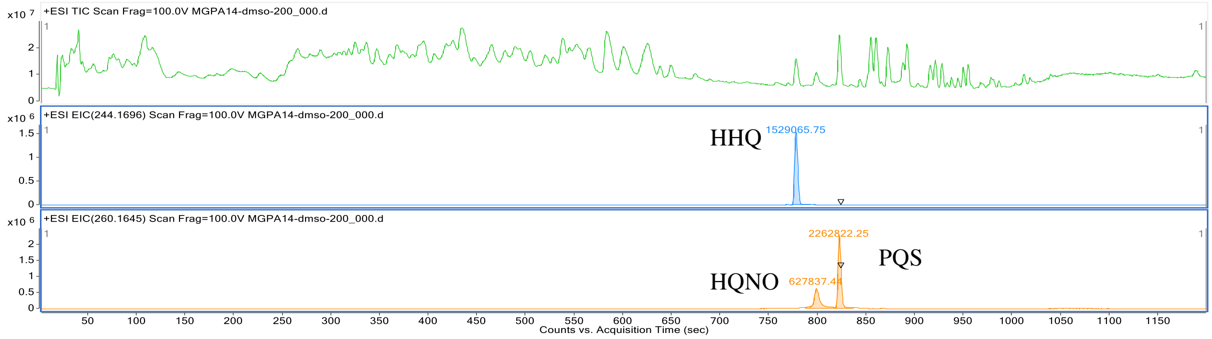

c. HHQ

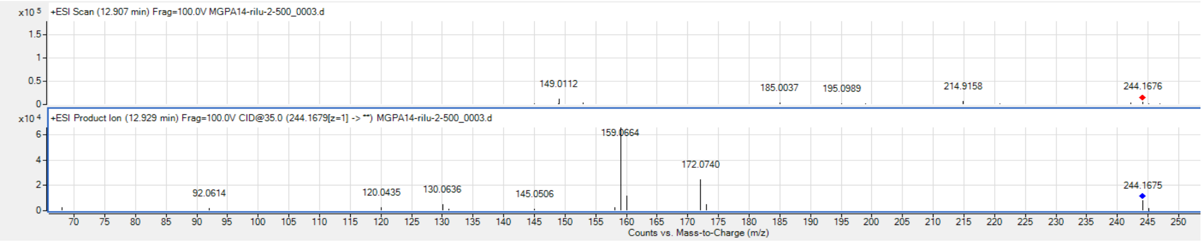

d. HQNO

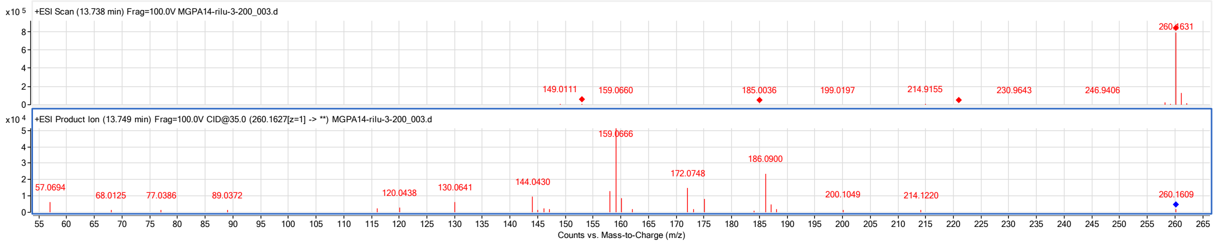

e. PQS

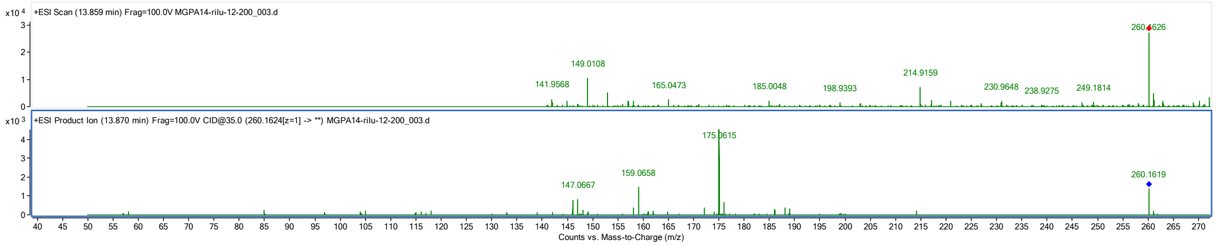

Figure S7. Quinolones (a) were analyzed by mass spectrometry and their identities confirmed by LC-MS elution time (b) and mass spectral data, including fragmentation patterns (MS^2) [**HHQ** (c), **HQNO** (d), **PQS** (e)] as previously published by Rahme *et al.*[4]

Biofilm assays

P. aeruginosa PA14 strain was grown overnight (~16 h) in LB media at 37 °C and 200 RPM. The overnight culture was diluted 1:100 in fresh LB media. 200 μL of this culture was then used to inoculate each well of a microtiter plate, to which **Rilu**-inhibitors were added in DMSO to final concentration of 200 μM or 500 μM (for the control experiments, same amount of DMSO was added). Eight wells per plate were used for each sample. These cultures were grown for 24 h at 37 °C without shaking. After growth, the planktonic cultures were carefully removed to a new microtitre plate to measure the OD_{620} values. The adhered cells were washed carefully three times with sterile PBS buffer, and then the cells were fixed with MeOH for 20 min. After removing the MeOH, the wells were allowed to dry for 10 min, followed by addition of 0.1% crystal violet. After 15 min of incubation, the stain was carefully removed without touching the rest of the plate. The excess stain was washed away 3 times from the wells with distilled water and the plate was allowed to dry for ~1 h. Finally, the adhered stain was redissolved in 30% acetic acid solution (for 1 h) and absorbance was measured at 595 nm. Assays were repeated in triplicate.

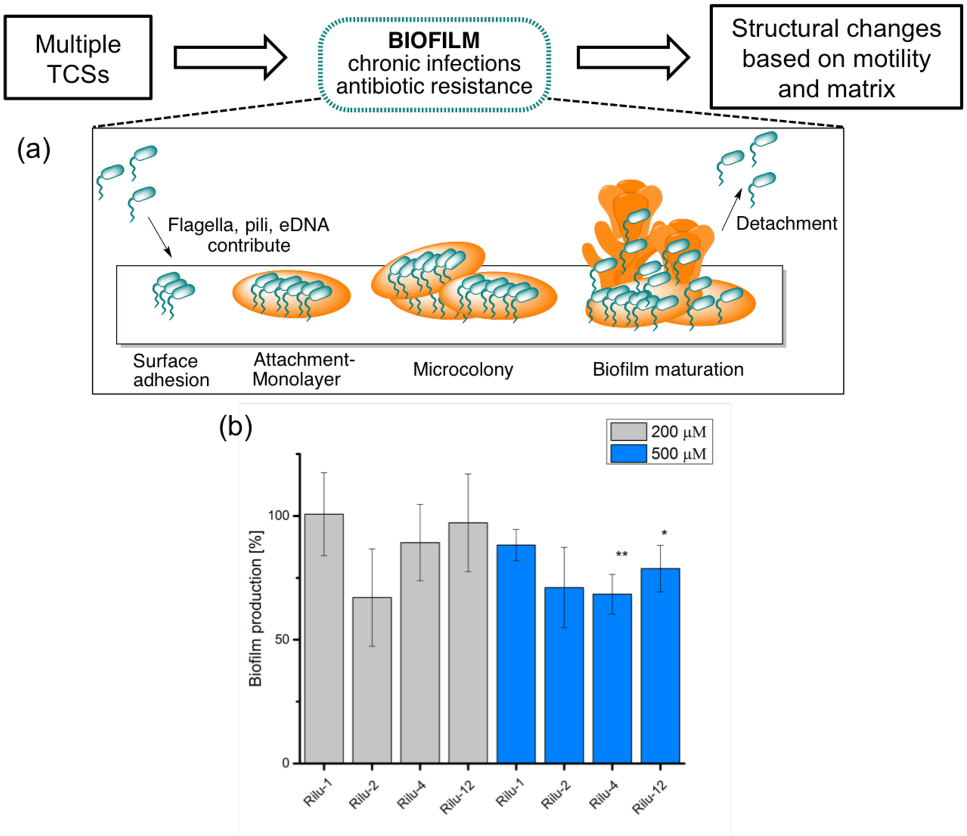

Figure S8. (a) Different stages of biofilm formation. (b) Biofilm formation assessment. All values are plotted relative to a DMSO-treated control and normalized to the final cell density (OD_{620}). Error bars represent the standard error of three independent experiments. Statistical analysis performed with non-parametric one-way ANOVA ($\alpha = 0.05$; $^{**}p < 0.02$, $^{*}p < 0.05$).

Rapid attachment assays[5]

P. aeruginosa PA14 strain was grown overnight (~16 h) in LB media at 37 °C and 200 RPM. The overnight cultures were washed and diluted in LB medium to an OD_{620} value of 1.0. 100 μL of this suspension was used to inoculate each well of a microtiter plate in presence of **Rilu**-inhibitors in DMSO at 200 μM or 500 μM (for the control experiments, same amount of DMSO was added). Eight wells per plate were used for each sample. Cells were allowed to adhere for 60 min at 37 °C and 200 RPM. After the planktonic cells were removed, staining with crystal violet was carried out, as described in the biofilm assay procedure. Assays were repeated in triplicate.

Swarming assays and quantification of rhamnolipids in swarm-plates

We followed the revised protocol developed by Trembley *et al.* for *P. aeruginosa* swarm assays.[6] Plates consisted of modified M9 medium [20 mM NH_4Cl; 12 mM Na_2HPO_4; 22 mM KH_2PO_4; 8.6 mM NaCl; 1 mM $MgSO_4$; 1 mM $CaCl_2$.$2H_2O$; 11 mM dextrose; 0.5% casamino acids (Difco)] solidified with Bacto-agar (Difco). The M9 media without $MgSO_4$ and $CaCl_2$•$2H_2O$ was autoclaved, which were added after cooling the media ~60 °C. The pH was adjusted after autoclaving with HCl and NaOH (pH = 7.5). Then, a 1-in-100 dilution of filter-sterile stock containing 1 mg/mL Nile red (Sigma Aldrich) dissolved in 85% ethylene glycol and added (prepared the day of use to limit photoinactivation).[7] Twenty mL of this media was poured into Petri dishes, along with the **Rilu**-inhibitors in DMSO at final concentrations, 125 μM or 200 μM (for the control experiments, same amount of DMSO was added). Plates were allowed to dry for 60 min, with the first 20 min under UV light to ensure plates were sterile following pH adjustment. *P. aeruginosa* PA14 strain was grown overnight (~16 h) in LB media at 37 °C and 200 RPM. Five μl of bacteria were spotted at the center of each plate, which were incubated at 30 °C in the dark and ~20% humidity. The plates were intermittently taken out for fluorescence scanning [scan settings: GE Typhoon Variable Mode Imager 9500, using 532-nm laser with DY-520XL (λex: 520 nm, λem: 664 nm)]. The scans were visualized and integrated density measurements of whole-plate fluorescence was measured using ImageJ software. Images of these plates were also taken with a Nikon D5200 DSLR camera, which were processed in Adobe Photoshop Lightroom 5 (Mac version) for phase-contrast.

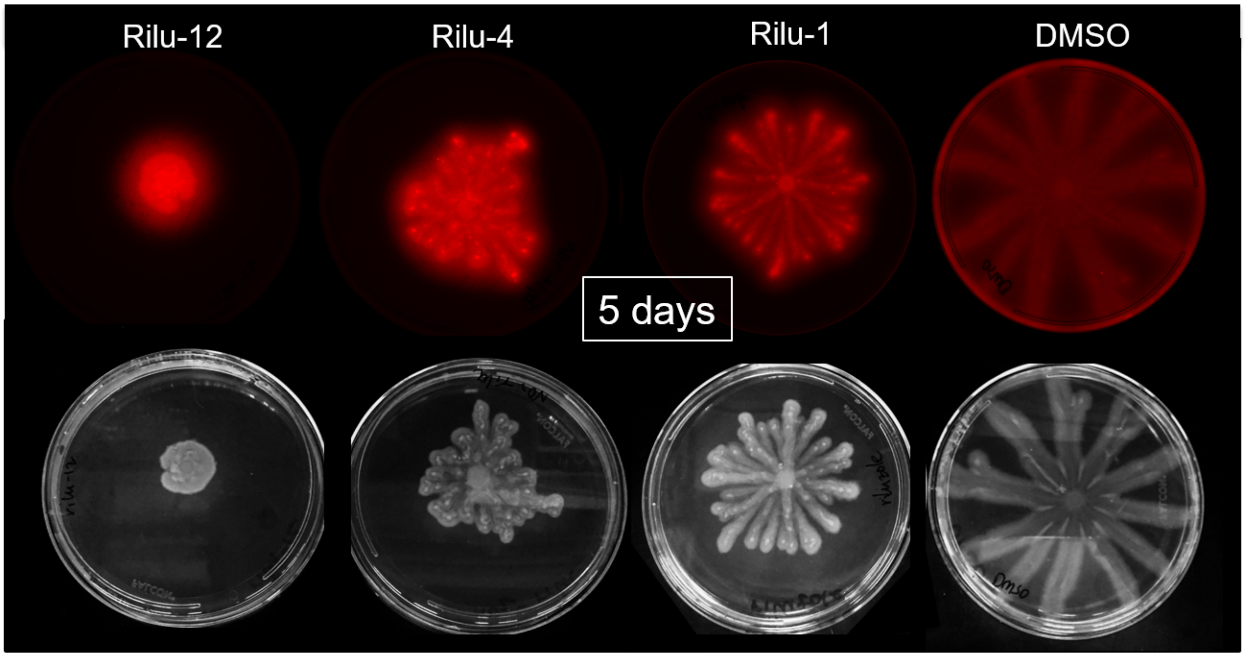

Figure S9. Swarming motility of PA14 in the presence of **Rilu**-inhibitors at 200 μM after 5 days. The top images are fluorescence scans for detecting Nile Red and the bottom images were taken with a digital camera.

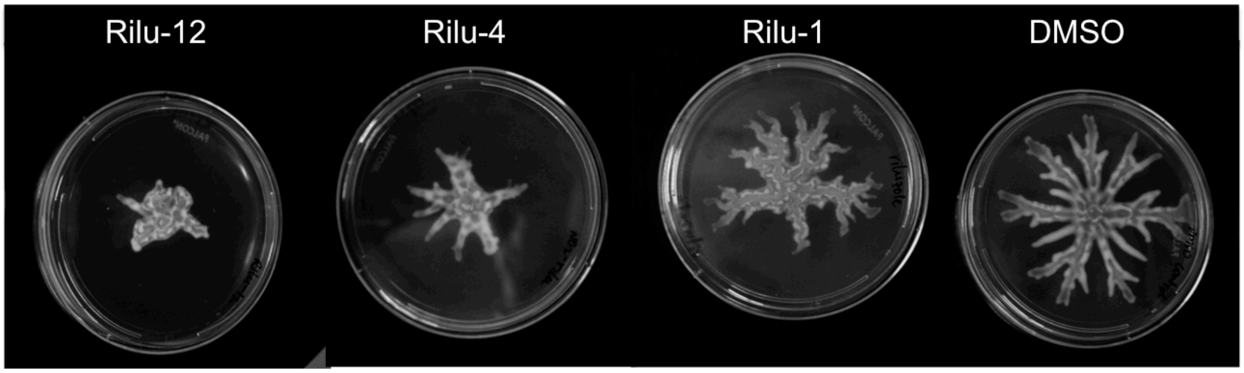

Figure S10: Images of the swarm plates with inhibitors at 125 μM or DMSO after 48 h.

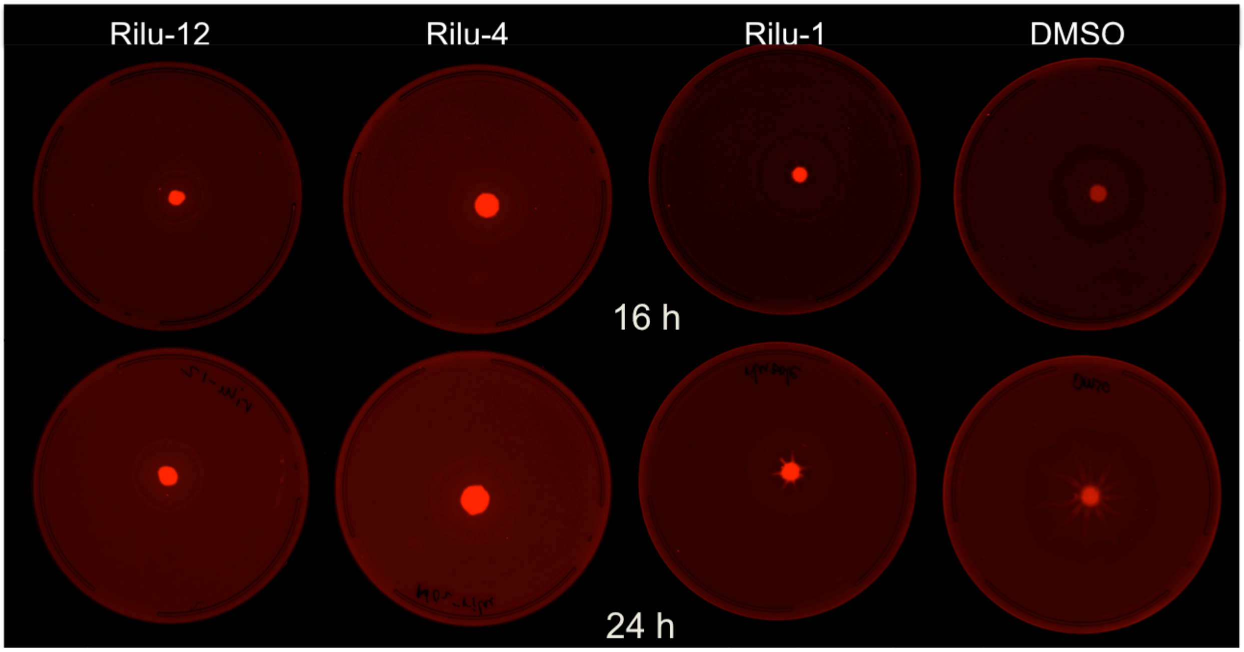

Figure S11: Fluorescence scans of the swarm plates with inhibitors at 200 μM or DMSO at 16 h and 24 h.

RNA isolation, Reverse Transcriptase -qPCR experiments

P. aeruginosa PA14 strain was grown overnight (~16 h) in LB media at 37 °C and 200 RPM. The overnight culture was diluted 1:100 in fresh LB media. Four mL of this culture was then taken in a 15 mL borosilicate glass tube, to which **Rilu**-inhibitors in DMSO were added to a final concentration of 200 μM or 500 μM (for the control experiments, same amount of DMSO was added). These cultures were then grown for 9 h (late exponential phase) at 37 °C and 200 RPM, OD_{620} ~0.7. Total RNA was extracted from ~2 mL cell pellet using an RNAEasy Mini kit (Qiagen), according to the manufacturer's instructions, including exogenous DNase treatment with Ambion Turbo-DNAse Kits. RNA integrity was confirmed by native gel electrophoresis and the quantities were measured using a nanophotometer. cDNA was generated using the DNA-free total RNA using (Applied Biosystems High Capacity cDNA Reverse Transcription Kits) random-primed reverse transcriptase reaction following the manufacturer's protocol. The cDNA was used as template for quantitative PCR (BioRad MyiQ2 dual-color system) using the SYBR Green detection system (BioRad) on white 96-well PCR plates. Melt curves were obtained for every run, and any wells with multiple peaks were excluded from analysis. Signal was standardized to 2 housekeeping genes, *recA, rpoD* using the $\Delta\Delta C_T$ method equation:[8] Relative expression = 2(C_T standard –C_T sample), where C_T (cycle time) was determined automatically by the Real Time iQ5 PCR software (BioRad). Primers (Integrated DNA Technologies) for RT-qPCR were designed using NCBI Primer-Blast software. Criteria for primer design were a melting temperature = 59–61 °C; primer size = 18–22 bp; product size = 80–130 bp; product Tm = 60– 90 °C. Samples were assayed in triplicate and statistical analyses was done with one-way ANOVA (Origin 9.1 software).

Table S5. List of primer pairs used in qPCR

Target gene	Forward	Reverse
**PA14_17530 (*recA)***	TCACCGGCAATATCAAGAAC	CGAGGCGTAGAACTTCAGTG
**PA14_07520 (*rpoD*)**	CAAGATCCGCAAGGTACTGA	GGTGGAGTCCTCGATGAAAT
**PA14_45940 *(lasI)***	GCCCCTACATGCTGAAGAAC	TCCAGAGTTGATGGCGAAA
**PA14_45960 (*lasR)***	TCAAGTGGAAAATTGGAGTGG	TCGT AGTCCTGGCTGTCCTT
**PA14_51430 (*pqsA)***	ACCTGACCGAGGTTCTGTTC	TGGCCTGGGAGAGAATGTAG
**PA14_51340 (*pqsR*)**	TCGTTCTGCGATACGGTGAG	TTGATCGTCGCCAGGCTATC
**PA14_19130 (*rhlI*)**	AGTTCGACCATCCGCAAA	CTGCACAGGTAGGCGAAGA
**PA14_19120 (*rhlR*)**	GAGCGATACCAGATGCAGAAC	GCTCCAGACCACCA TTTCC
**PA14_30650 (*gacA*)**	TCGTCCTGATGGACGTGAAG	CTCTTCGCAGACGGTGACTA
**PA14_06875 (*rsmY*)**	GAAGCGCCAAAGACATACGG	GCAGACCTCTATCCTGACATCC

**PA14_52570 (*rsmA*)**	ACGGTACTGGGTGTCAAAGG	GCTGGTAAATTTCCTCCCGGT
**PA14_50220 (*fleQ*)**	GCAACAAGACGCAGAACGTC	AGAGGTCTTCGCGGAAAGTG
**PA14_58730 (*pilA*)**	CAAACCGAGAAGGTCGGACT	AAGCATCTTGCATGCCAACC

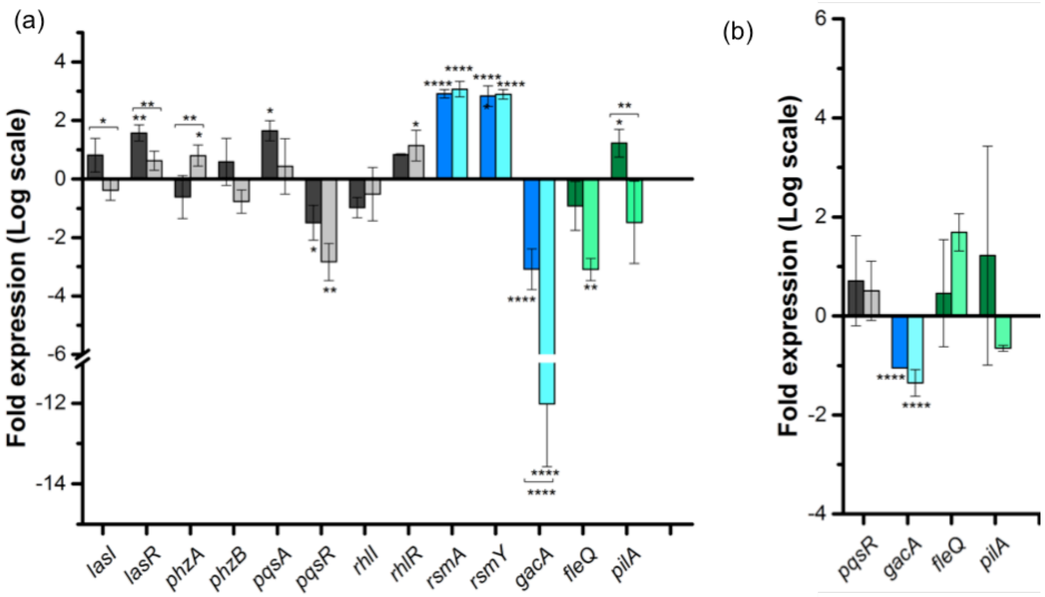

Figure S12. qPCR analysis of various genes in PA14 upon exposure to (a) **Rilu-4** and (b) **Rilu-7**, fold expression is shown in log 2 scale. Genes are colored based on three categories: QS-related (grey), TCS GacSA-related (blue) and motility-related (green). Darker and lighter bars (grey, blue and green) are measurements for **Rilu-4** or **7** at 200 µM and 500 µM, respectively. Error bars represent the standard error of three independent experiments (n = 3). Statistical analysis performed with non-parametric one-way ANOVA (α= 0.05; ****p < 0.0001; ***p < 0.001; **p < 0.02, *p < 0.05). Asterisks closest to the bars denote the significance of the difference between inhibitor-treated samples and the DMSO-treated sample, while the asterisks outside the bracket denote the significance of the difference between the two inhibitor concentrations.

Quantification of rhamnolipids in liquid cultures

Rhamnolipid was quantified according to the method of Welsh *et al*. with minor modifications.[9] Briefly, *P. aeruginosa* PA14 strain was grown overnight (~16 h) in LB media at 37 °C and 200 RPM. This cultures was diluted 1:100 into fresh Minimal Medium (49.3 mM Na_2HPO_4, 50 mM KH_2PO_4, 4.8 mM $MgSO_4$, 7.6 mM $(NH_4)_2SO_4$, 0.6 mM $CaCl_2$, 25 μM $FeSO_4$, 0.162 μM $(NH_4)_6Mo_7O_{24}$, 38 μM $ZnSO_4$, 14 μM $MnCl_2$, 1.6 μM $CuSO_4$, 0.86 μM $CoCl_2$, 1.9 μM boric acid, 5.5 μM $NiCl_2$, 6.72 μM EDTA, 0.6% glycerol). 4 mL of this culture was taken in a 15 mL borosilicate glass tube, to which **Rilu**-inhibitors in DMSO were added to a final concentration of 200 μM (for the control experiments, same amount of DMSO was added). These cultures were grown for 20 h at 37 °C and 200 RPM. The final OD_{620} was measured, and the cells were pelleted at 5000 *g* for 5 min. 1 mL of the supernatant was taken and extracted twice with 1 mL of diethyl ether. The pooled organic extracts were dried over anhydrous magnesium sulfate, evaporated to dryness and then reconstituted in 200 μL deionized water. Then, 50 μL of this extract was diluted into 450 μL of a solution of 0.19% (w/v) orcinol in 50% (v/v) concentrated H_2SO_4. The tubes were briefly vortexed and incubated in an 80 °C heating block for 45 min. After briefly cooling to room temperature, 200 μL of the resulting solution was transferred to a clear 96-well microtiter plate and the absorbance at 421 nm measured. Data were normalized to the final OD_{620} values and plotted relative to a DMSO control.

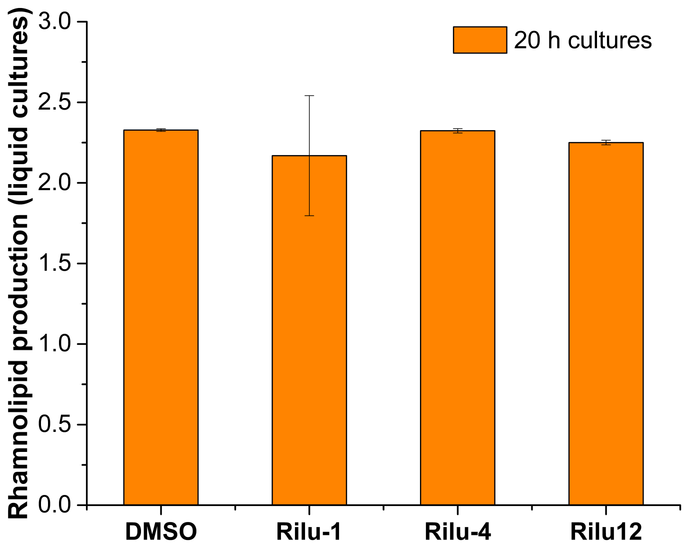

Figure S13. Rhamnolipid production of PA14 in liquid minimal media. Error bars represent the standard error of three independent experiments (n = 3).

References:

1. K. E. Wilke, S. Francis and E. E. Carlson, *JACS*, 2012, **134**, 9150-9153.
2. C. A. Schneider, W. S. Rasband and K. W. Eliceiri, *Nat. Methods*, 2012, **9**, 671-675.
3. K. E. Wilke, S. Francis and E. E. Carlson, *ACS Chem. Bio.*, 2015, **10**, 328-335.
4. F. Lépine, S. Milot, E. Déziel, J. He and L. G. Rahme, *J. Am. Soc. Mass. Spectrom.*, 2004, **15**, 862-869.
5. A. Neidig, A. T. Yeung, T. Rosay, B. Tettmann, N. Strempel, M. Rueger, O. Lesouhaitier and J. Overhage, *BMC Microbiol.*, 2013, **13**, 77.
6. J. Tremblay and E. Deziel, *J. Basic Microbiol.*, 2008, **48**.
7. J. D. Morris, J. L. Hewitt, L. G. Wolfe, N. G. Kamatkar, S. M. Chapman, J. M. Diener, A. J. Courtney, W. M. Leevy and J. D. Shrout, *Appl. Environ. Microbiol.*, 2011, **77**, 8310-8317.
8. M. W. Pfaffl, *Nucleic Acids Res.*, 2001, **29**, e45-e45.
9. M. A. Welsh, N. R. Eibergen, J. D. Moore and H. E. Blackwell, *JACS*, 2015, **137**, 1510-1519.
